# Pangenomes suggest ecological-evolutionary responses to experimental soil warming

**DOI:** 10.1128/msphere.00059-25

**Published:** 2025-03-19

**Authors:** Mallory J. Choudoir, Achala Narayanan, Damayanti Rodriguez-Ramos, Rachel Simoes, Alon Efroni, Abigail Sondrini, Kristen M. DeAngelis

**Affiliations:** 1 Department of Microbiology, University of Massachusetts Amherst, Amherst, Massachusetts, USA; 2 Department of Plant and Microbial Biology, North Carolina State University, Raleigh, North Carolina, USA; 3 Department of Plant and Microbial Biology, University of Minnesota, St. Paul, Minnesota, USA; 4 Department of Bacteriology, University of Wisconsin Madison, Madison, Wisconsin, USA; E O Lawrence Berkeley National Laboratory, Berkeley, California, USA

**Keywords:** climate change, soil, adaptation, pangenome, traits, CAZyme, codon usage bias

## Abstract

Below-ground carbon transformations that contribute to healthy soils represent a natural climate change mitigation, but newly acquired traits adaptive to climate stress may alter microbial feedback mechanisms. To better define microbial evolutionary responses to long-term climate warming, we study microorganisms from an ongoing *in situ* soil warming experiment where, for over three decades, temperate forest soils are continuously heated at 5°C above ambient. We hypothesize that across generations of chronic warming, genomic signatures within diverse bacterial lineages reflect adaptations related to growth and carbon utilization. From our bacterial culture collection isolated from experimental heated and control plots, we sequenced genomes representing dominant taxa sensitive to warming, including lineages of Actinobacteria, Alphaproteobacteria, and Betaproteobacteria. We investigated genomic attributes and functional gene content to identify signatures of adaptation. Comparative pangenomics revealed accessory gene clusters related to central metabolism, competition, and carbon substrate degradation, with few functional annotations explicitly associated with long-term warming. Trends in functional gene patterns suggest genomes from heated plots were relatively enriched in central carbohydrate and nitrogen metabolism pathways, while genomes from control plots were relatively enriched in amino acid and fatty acid metabolism pathways. We observed that genomes from heated plots had less codon bias, suggesting potential adaptive traits related to growth or growth efficiency. Codon usage bias varied for organisms with similar 16S *rrn* operon copy number, suggesting that these organisms experience different selective pressures on growth efficiency. Our work suggests the emergence of lineage-specific trends as well as common ecological-evolutionary microbial responses to climate change.

**IMPORTANCE:**

Anthropogenic climate change threatens soil ecosystem health in part by altering below-ground carbon cycling carried out by microbes. Microbial evolutionary responses are often overshadowed by community-level ecological responses, but adaptive responses represent potential changes in traits and functional potential that may alter ecosystem function. We predict that microbes are adapting to climate change stressors like soil warming. To test this, we analyzed the genomes of bacteria from a soil warming experiment where soil plots have been experimentally heated 5°C above ambient for over 30 years. While genomic attributes were unchanged by long-term warming, we observed trends in functional gene content related to carbon and nitrogen usage and genomic indicators of growth efficiency. These responses may represent new parameters in how soil ecosystems feedback to the climate system.

## INTRODUCTION

Humanity faces an existential threat of accelerating soil loss due to habitat degradation and climate change (1), but the consequences of microbial adaptation to climate change remain insufficiently explored. Microbes are both impacted by climate change and are themselves agents of global change (2–4). Soils are home to the most diverse microbial communities on the planet, and soil health is predicated on microbial diversity and activity (5, 6). Ultimately, how soil microbes respond to and evolve with changing soil habitats will determine terrestrial ecosystem function under future climate change scenarios.

Rising soil temperatures due to climate change are altering microbial global nutrient cycles in complex ways (7, 8). Worse, chronic warming may disrupt the microbial mechanisms governing carbon fluxes between the biosphere and atmosphere, mechanisms which may provide a natural attenuation of the self-reinforcing feedbacks to climate (9). Soils release considerable amounts of atmospheric carbon dioxide (CO_2_) through microbial decomposition of organic matter (10) but are also the largest terrestrial reservoir of global carbon (11, 12). Microbes mediate key roles in carbon fluxes between terrestrial and atmospheric systems, evidenced by the fact that incorporating microbial processes into Earth system models improves global carbon pool projections (13–15). Temperate forests actively sequester 0.7 Pg C year^−1^, but the potential for forests to mitigate climate change by accumulating stable, persistent carbon is uncertain (16–18). The fate of terrestrial forest carbon pools largely depends on how microbes respond to global change factors.

Long-term field warming experiments allow us to gain insights into what soils could look like in a warming world. For over three decades, temperate forest soils at the Harvard Forest Long-Term Ecological Research (LTER) site have been continuously heated *in situ* by 5°C above ambient temperatures (19). After 26 years, warming degraded soil organic carbon quantity and quality, increased net CO_2_ emissions, and altered microbial activity in heated versus control plots (20, 21). Though fungal biomass and community structure were drastically changed with warming (22, 23), bacterial biomass and community structure remained relatively unperturbed by warming, with significant changes observed only after 20 years in the organic horizon (24). Finally, bacteria isolated from the heated Harvard Forest plots showed greater potential to degrade lignin and other plant polymers compared to those isolated from control plots (25, 26), suggesting that traits related to substrate use may explain long-term dynamics of climate stress on microbial systems.

Both ecological and evolutionary mechanisms shape how environmental microbes respond to climate change, but lineage-specific evolutionary responses are often overshadowed by community-level ecological responses. Ecological filtering predominantly shapes community assembly across different habitats (27, 28). Regional pools of diversity, high dispersal rates, and a propensity for dormancy also dictate how microbial communities change, or not, in response to environmental disturbance (29, 30). Canonically, we expect to observe similar ecological forces shaping microbial community responses to soil warming. However, due to large population sizes and short generation times, co-occurring ecological and evolutionary forces can act on more similar time scales for natural microbial populations across years or decades (31). We do not know the cascading impacts of local or regional microbial adaptation to climate change on soil ecosystem functions (32). Theoretical carbon models need to account for ecological and evolutionary feedbacks across spatiotemporal scales within these dynamic selective landscapes (33, 34).

Based on our previous works, ecological filtering in heated plots likely selects for microbes able to degrade more recalcitrant carbon substrates (25, 26) in response to warming-induced decreases in carbon quality and quantity (20). Further, a recent study found increased relative abundances of functional genes involved in lignin degradation in metagenomes from heated compared to control soils (35). However, evolutionary processes may also contribute to differences in carbon metabolism between treatment plots. Long-term warming causes thermal acclimation of soil respiration (36, 37), though this effect may be seasonal (38). Microbial thermal adaptation likely involves physiological trade-offs altering growth rate and efficiency (39). More broadly speaking, life history trade-offs between growth efficiency, resource acquisition, and stress tolerance shape organismal fitness (40) linking population and community-level trait evolution.

We hypothesize that Harvard Forest microbial lineages have evolved adaptive traits related to carbon substrate metabolism and growth efficiency across generations of chronic soil warming. To maximize our ability to identify genomic signatures of adaptation, we used comparative pangenome approaches on collections of closely related bacterial taxa across multiple independent lineages spanning Actinobacteria, Alphaproteobacteria, and Betaproteobacteria. We explored genomic attributes, patterns of functional gene content presence and absence, and codon usage between heated and control genomes to identify putative adaptive traits that have emerged in this system since the onset of the long-term warming experiment.

## MATERIALS AND METHODS

### Experimental site and bacterial culture collection

We isolated bacteria from the Harvard Forest long-term soil warming experiment plots between 2013 and 2020 using a range of enrichment conditions (26, 41; Table S1) targeting taxa of interest. Briefly, we sampled soils (0–10 cm) using a stainless steel soil corer, manually separated mineral and organic horizons, and sieved (2 mm). We plated soil dilutions onto solid media (Table S1) and incubated them at room temperature for 2–8 weeks. Following the initial enrichment, colonies were picked and streaked to isolation on 10% tryptic soy agar (TSA) or Reasoner’s 2A (R2A), genotyped with full-length 16S rRNA gene sequences (27F-AGAGTTTGATCMTGGCTCAG and 1492R-TACGGYTACCTTGTTACGACTT [42]), and cryopreserved in 20% glycerol at −80°C.

### Genomic DNA (gDNA) extraction and whole genome sequencing

We inoculated colonies into 10 mL 10% tryptic soy broth, grew them to stationary phase at 28°C with shaking, and pelleted the cells by centrifugation. We extracted high molecular weight gDNA from cell pellets using either the DNeasy Blood & Tissue Kit (Qiagen, Germantown, MD, USA) or a modified CTAB-lysozyme extraction protocol (43) (with phenol-chloroform instead of chloroform and extended centrifugation times). We confirmed average fragment size of 30–50 kb with gel electrophoresis and quantified DNA using a Qubit Fluorometer (Thermo Fisher Scientific, Waltham, MA, USA) to verify at least 1 µg of gDNA. We determined DNA quality using a NanoDrop Spectrophotometer (Thermo Fisher Scientific, Waltham, MA, USA) with absorbance ratios 260/280 within 1.8–2.0 and 260/230 within 2.0–2.3.

A subset of genomes was sequenced and assembled at the DOE Joint Genome Institute (JGI) (Berkeley, CA, USA) using either PacBio RS (Pacific Biosciences, Menlo Park, CA, USA) or NovaSeq S4 (Illumina, San Diego, CA, USA) (Table S1). A subset of betaproteobacterial genomes was sequenced at SeqCenter (Pittsburgh, PA, USA) using NextSeq 2000 (Illumina, San Diego, CA, USA). All remaining genomes were sequenced in-house using ONT MinION (Oxford Nanopore Technologies, Oxford, UK).

We prepared ONT MinION sequencing libraries using the Ligation Sequencing Kit SQK-LSK-109 (ONT, Oxford, UK). We multiplexed six to eight genomes per sequencing run using the Native Barcoding Expansion Kit EXP-NBD104 (ONT, Oxford, UK). We skipped the Covaris g-TUBE shearing step to target long fragment DNA. Starting with 1 µg of DNA, we prepped samples with the NEBNext FFPE DNA Repair Mix and NEBNext Ultra II End Repair/dA-Tailing kits (New England Biolabs, Ipswich, MA, USA) and performed DNA cleanup with Ampure XP beads (Beckman Coulter, Pasadena, CA, USA). We pooled approximately 150 ng of each barcoded sample for a final library of 700–1,000 ng, and adapters were ligated to the sample with Blunt/TA ligase (New England Biolabs, Ipswich, MA, USA). The long fragment buffer was used in an extended 10-min incubation at 37°C to enrich for high molecular weight DNA. We primed the flow cell using the Flow Cell Priming Kit (ONT, Oxford, UK), mixed, and loaded approximately 15 fmols of the pooled library, sequencing buffer, and loading beads. Sequencing runs were completed after 72 h, and we ran high-accuracy base calling on the raw fast5 data with the ONT software Guppy v4.5.4 or v6.1.1 (Oxford Nanopore Technologies, 2021).

### ONT MinION genome assembly

For all genomes sequenced using ONT MinION, we performed either *de novo* long-read assembly or hybrid short- and long-read assembly. We used Filtlong (https://github.com/rrwick/Filtlong) to sub-sample high-quality reads (–min_length 1000 –min_mean_q 85) to approximately 40× estimated genome coverage and Flye v2.8.1 (44) for *de novo* assembly. We used Racon v1.4.2 (45) to generate a consensus assembly and performed final polishing with medaka v1.2.1 (Oxford Nanopore Technologies, 2018). For the subset of betaproteobacterial genomes with both short- and long-read sequence data, we used unicycler (46) to perform the hybrid assembly. Quast (47) and CheckM (48) assessed the genome assembly quality. We considered a genome assembly high quality if it had less than 5% contamination and at least 90% estimated completeness (Table S3) (49).

### Genome annotation

We conducted genomic analyses within the anvi’o v8 software ecosystem (50). Genome assemblies were imported into anvi’o with the program anvi-gen-contigs-database, and open reading frames were identified using Prodigal v2.6.3 (51). The program anvi-run-kegg-kofams assigned KEGG Ortholog (KO) functions to protein-coding genes (52), and the program anvi-estimate-metabolism reconstructed the metabolic capabilities of each isolate genome (53). Finally, we predicted catalytic and carbohydrate-binding module domains of carbohydrate-active enzymes (CAZymes) using anvi-run-cazymes to run Hidden Markov Model (HMM) searches against dbCAN HMMdb v11 (54, 55).

### Pangenome construction

We constructed pangenomes for each clade with the program anvi-pan-genome. Protein comparisons were computed with DIAMOND (56) with minbit heuristic = 0.5 (default value) to eliminate weak matches. Finally, the Markov cluster algorithm (MCL), described by Dongen and Abreu-Goodger (57), was used with an inflation value of 6, 8, or 10 depending on the phylogenetic relationships of the clade (Table 1). This approach identified shared gene clusters (GCs) or collections of similar genes. Core GCs are defined as GCs present in all genomes. Accessory GCs are those present in at least two but not all genomes. Finally, singletons are GCs unique to a single genome. Within each clade, the program anvi-compute-genome-similarity computed pairwise average nucleotide identity (ANI) using the software program pyANI and ANIb method (58).

**TABLE 1:** Pangenome characteristics for the given phyla and clades

Characteristic^*a*^	Actinobacteria, clade Kitasatospora	Alphaproteobacteria		Betaproteobacteria	
		Bradyrhizobium	Rhizobium	Paraburkholderia	Ralstonia
Genomes, *n*	28	15	14	16	18
Genome ANI	0.795–0.999	0.788–0.999	0.902–0.999	0.799–0.999	0.955–0.999
16S rRNA gene similarity	0.977–1.00	0.971–1.00	0.997–1.00	0.980–1.00	1.00
MCL inflation	6	6	10	8	10
Total GCs	30,917	26,027	7,961	24,707	5,157
Core GCs	2,085	2,580	3,732	2,719	4,518
p_Core	0.067	0.099	0.469	0.11	0.876
Accessory	18,691	10,929	3,232	10,645	463
p_Accessory	0.605	0.420	0.406	0.43	0.090
Singletons	10,141	12,518	997	11,343	176
p_Singletons	0.328	0.481	0.125	0.46	0.034

^*a*^ *n*, number of genomes in each pangenome; ANI, genome wide average nucleotide identity; MCL inflation, Markov cluster algorithm inflation values used for generating gene clusters; GCs, gene clusters; p_Core, portion of GCs that belong to the core gene pool; Accessory, number of accessory GCs; p_Accessory, portion of GCs that belong to the accessory gene pool; Singletons, number of singletons GCs; p_Singletons, portion of GCs that are singletons.

### Phylogenetic analysis

Phylogenetic relationships were reconstructed from conserved genes. 16S ribosomal RNA (rRNA) genes and single-copy ribosomal protein genes were identified with HMM searches using the command anvi-run-hmms with anvi’o databases Ribosomal_RNA_16S and Bacteria_71. Nucleotide sequences for 16S rRNA genes and 39 concatenated ribosomal protein genes were extracted with the program anvi-get-sequences-for-hmm-hits and aligned with MUSCLE (59). Maximum likelihood (ML) trees were built using the generalized time-reversible nucleotide substitution model with gamma-distributed rate heterogeneity among sites (GTRGAMMA) in RAxML-NG v1.2.2 (60), and bootstrap support was determined from 100 iterations. We used iTol (61) to visualize trees. Finally, we used the R package phytools to perform phylogenetic analysis of variance (ANOVA) with 1,000 simulations (62). We used BLASTn (63) to query isolate 16S rRNA gene sequences at >99% identity against partial 16S rRNA gene sequences (254 bp) of the dominant subset community (*n* = 155 OTUs, rank abundance) from a previous amplicon study conducted at the Harvard Forest warming experiment (24).

### Genome trait analyses

We used the program anvi-compute-functional-enrichment-in-pan and anvi-compute-metabolic-enrichment to identify individual GCs, functional annotations, and full metabolic pathway modules differently enriched in heated and control treatment groups (64). Some discussions of functional annotations were based on unadjusted *P*-value cutoffs as opposed to corrected *Q-*values. We chose to include these observations as potential trends, but we acknowledge that this may generate false positives and more robust conclusions require additional investigation. We used principal coordinate analysis of Bray–Curtis dissimilarities to visualize differences in CAZyme composition between clades and warming experiment treatments with the R package phyloseq (65) and permutational ANOVA to assess statistical differences following 999 permutations with vegan (66).

We used the R package coRdon (67) to calculate codon bias. First, we filtered genes with fewer than 80 codons before calculating codon usage metrics including the effective number of codons (ENC) (68) and the Measure Independent of Length and Composition (MILC) (69). For MILC measurements, we calculated genome-wide codon bias with respect to a subset of ribosomal proteins, which we speculated to be highly expressed genes. We used the R package effsize to calculate Cliff’s delta, a non-parametric effect size measurement (70), to determine the magnitude of codon bias measurement differences between treatment groups. All other statistical analyses were performed using R (71) statistical software, including FSA (72) for Dunn’s *post hoc* test of multiple comparisons.

## RESULTS AND DISCUSSION

### Harvard Forest soil warming experiment bacterial genome collection

We conducted comparative pangenomic analyses on bacteria isolated from a long-term warming field experiment to test our hypothesis that microbes adapt to local environmental change stressors. All bacterial isolates originated from a multi-decade climate manipulation experiment (21) at the Harvard Forest LTER site (Petersham, MA, USA). The Prospect Hill warming experiment was established in 1991 in a mixed deciduous forest stand. Heated cables buried 10 cm below the surface continuously maintain soil temperatures in heated plots at 5°C above the ambient temperatures of control plots (six replicate plots per treatment of 6 × 6 m^2^) (19). Between 2013 and 2020, we built a diverse culture collection of bacteria isolated from heated and control plots (see Materials and Methods and Table S1 for isolation details) targeting taxa dominant in soil communities and sensitive to long-term warming (24). Isolates for whole genome sequencing were selected from our culture collection based on their full-length 16S rRNA gene phylogeny and taxonomy.

**Fig 1:**
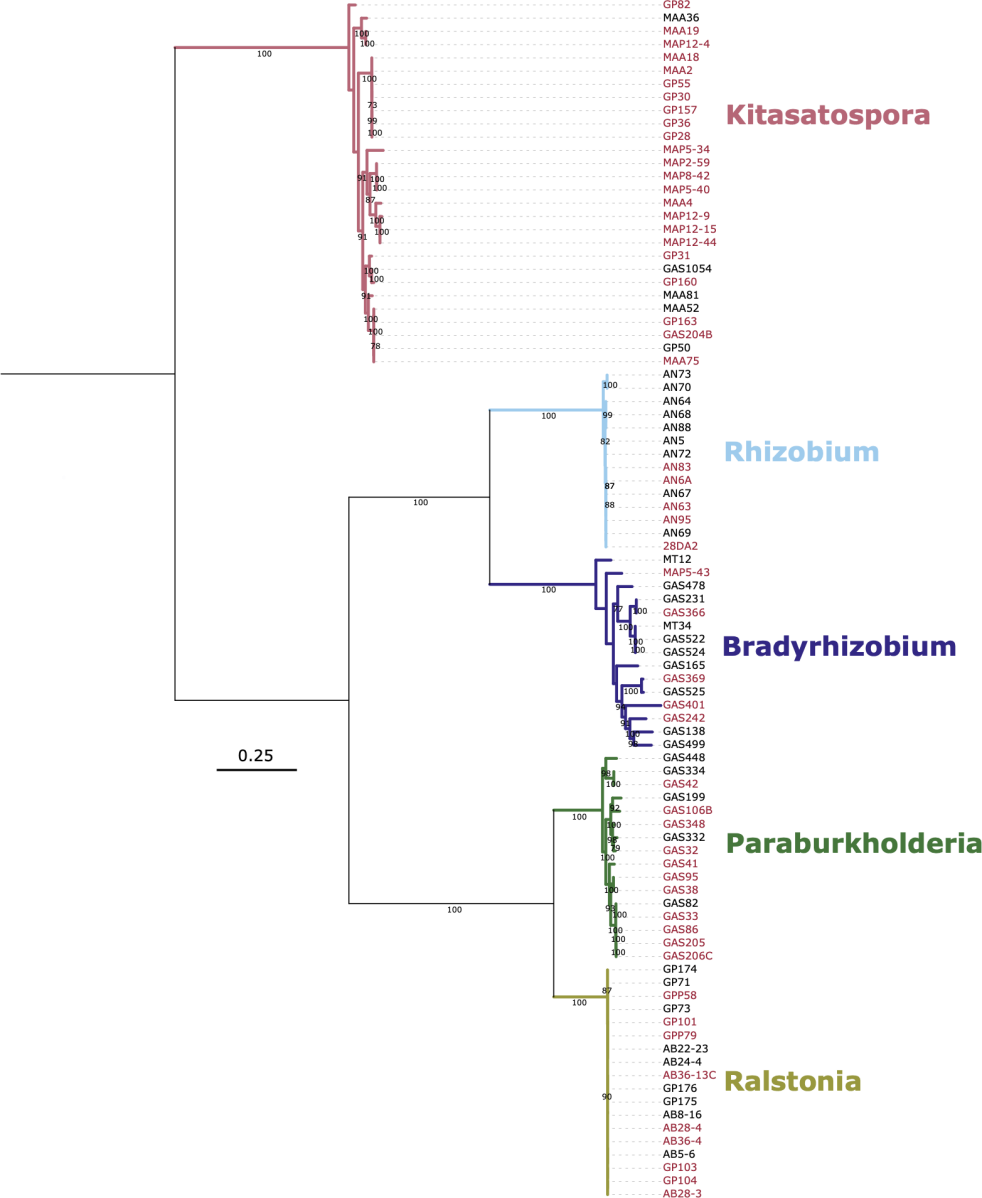
Ribosomal protein gene phylogeny. Tree shows the phylogenetic relationships between all 91 isolates (Table S1) constructed from 39 concatenated ribosomal protein gene nucleotide sequences derived from anvi’o HMM searches. Genome names are colored according to treatment group, with heated in red and control in black. Phylogenetic clades are also labeled and colored according to their pangenome group. Scale bar indicates nucleotide substitutions per site. Bootstrap values greater than 70 from 100 iterations are noted on tree branches. Tree is mid-rooted.

The final data set contained 91 genomes belonging to five clades spanning three phyla, including Actinobacteria *Kitasatospora* spp. (*n* = 28), Alphaproteobacteria *Bradyrhizobium* spp. (*n* = 15), Alphaproteobacteria *Rhizobium* spp. (*n* = 14), Betaproteobacteria *Paraburkholderia* spp. (*n* = 16), and Betaproteobacteria *Ralstonia* spp. (*n* = 18) (Fig. 1; Table S1). We chose these clades because we previously observed increased abundances of Actinobacteria and Alphaproteobacteria in Harvard Forest heated plots compared to control plots (24, 25). This aligns with a temperature sensitivity lab incubation study by Oliverio et al. (73) who found that across all sites, nearly all Actinobacteria taxa and approximately half of all Alphaproteobacteria taxa responded positively to warming. We also included Betaproteobacteria clades as a contrasting example of abundant soil taxa identified in our previous studies as less responsive to warming based on changes in community abundances. Partial 16S rRNA gene sequences from all *Bradyrhizobium* and some *Paraburkholderia* genomes mapped to dominant OTUs with higher relative abundances in heated and control plots, respectively (24). Importantly, partial 16S rRNA gene sequences from this previous study lack the resolution to distinguish strain-level variation (Fig. S1).

Actinobacteria, Alphaproteobacteria, and Betaproteobacteria are all globally dominant soil phyla (74, 75) and represent broad functional guilds delineated by varying life history strategies (76, 77). Though many ecosystem carbon models generally consider all bacteria as copiotrophic, operationally there exists a spectrum of growth efficiencies among phyla. Soil-dwelling Betaproteobacteria and Alphaproteobacteria are generally considered more copiotrophic with rapid growth and strong responses to labile carbon, while Actinobacteria typically embody more oligotrophic traits including slower growth and catabolic enzyme production (77, 78). Actinobacterial genomes also harbor abundant extracellular enzymes that break down plant-derived biomass in soils (79, 80).

The objective of our study was to identify genomic signatures at the organismal level that may link functional potential to adaptation. We generated high-quality draft genomes according to community standards (49) using a combination of sequencing platforms (Tables S1 to S3). Total contigs in assembled genomes varied considerably between sequencing platforms (Table S2). For *Paraburkholderia* spp., the number of assembled contigs strongly correlated with average gene length (Spearman’s ρ = −0.85, *P*-value < 0.0001) and genes per kb (Spearman’s ρ = 0.92, *P*-value < 0.0001). We also observed that assemblies with approximately >20 contigs had markedly fewer 16S rRNA gene copies than expected based on clade membership, so we omitted these genomes from downstream 16S rRNA gene analyses (see Table S2). However, there was no relationship between the number of assembled contigs and total genes or total GCs. We also assessed draft assembly quality by estimating completion based on the presence of bacterial single-copy genes, and all genomes have high estimated completion >94% (Tables S2 and S3). Given our focus on functional gene content, we concluded that these draft genomes were adequate for our study.

### Phylogeny and genomic attributes

Phylogenetic reconstruction of concatenated single-copy ribosomal protein genes showed five monophyletic clades with varying levels of intra-group relatedness (Fig. 1). All clades shared >97% similarity at 16S rRNA gene sequences (Table 1; Fig. S1) suggesting that pangenomes represent intra-species comparisons. However, genome-wide ANI ranged from 78% to 99% (Table 1) indicating that some groups represent more distant genus-level comparisons (81), while other groups span inter-species to intra-species comparisons (ANI ≥ 95%) (82) to nearly identical isolates (ANI > 99%). Our goal here is not to demarcate species or taxonomic boundaries, only to frame the phylogenetic and genomic relatedness of our genomes within the ongoing discussion of bacterial diversity.

To identify adaptive genomic traits associated with long-term soil warming that are shared among the clades, we first determined if broad genome attributes varied by warming treatment. While all genome traits differed significantly between clades, they did not vary by treatment within any of the clades (Kruskal-Wallis rank-sum test on the interaction between clade and treatment followed by *post hoc* pairwise Dunn test across intra-clade treatment groups, *P*-value > 0.1). Attributes evaluated included genome size, total number of genes, genes per kb, average gene length, G + C content, 16S rRNA gene copy number, total number of pangenome GCs, number of unique or singleton genes, and the total number of genes annotated as CAZymes (Table S2). When controlling for phylogeny, these attributes also did not vary by treatment (phylogenetic ANOVA, *P*-value > 0.1).

### Pangenome structure, gene content, and functional enrichment trends between warming treatments

**Fig 2:**
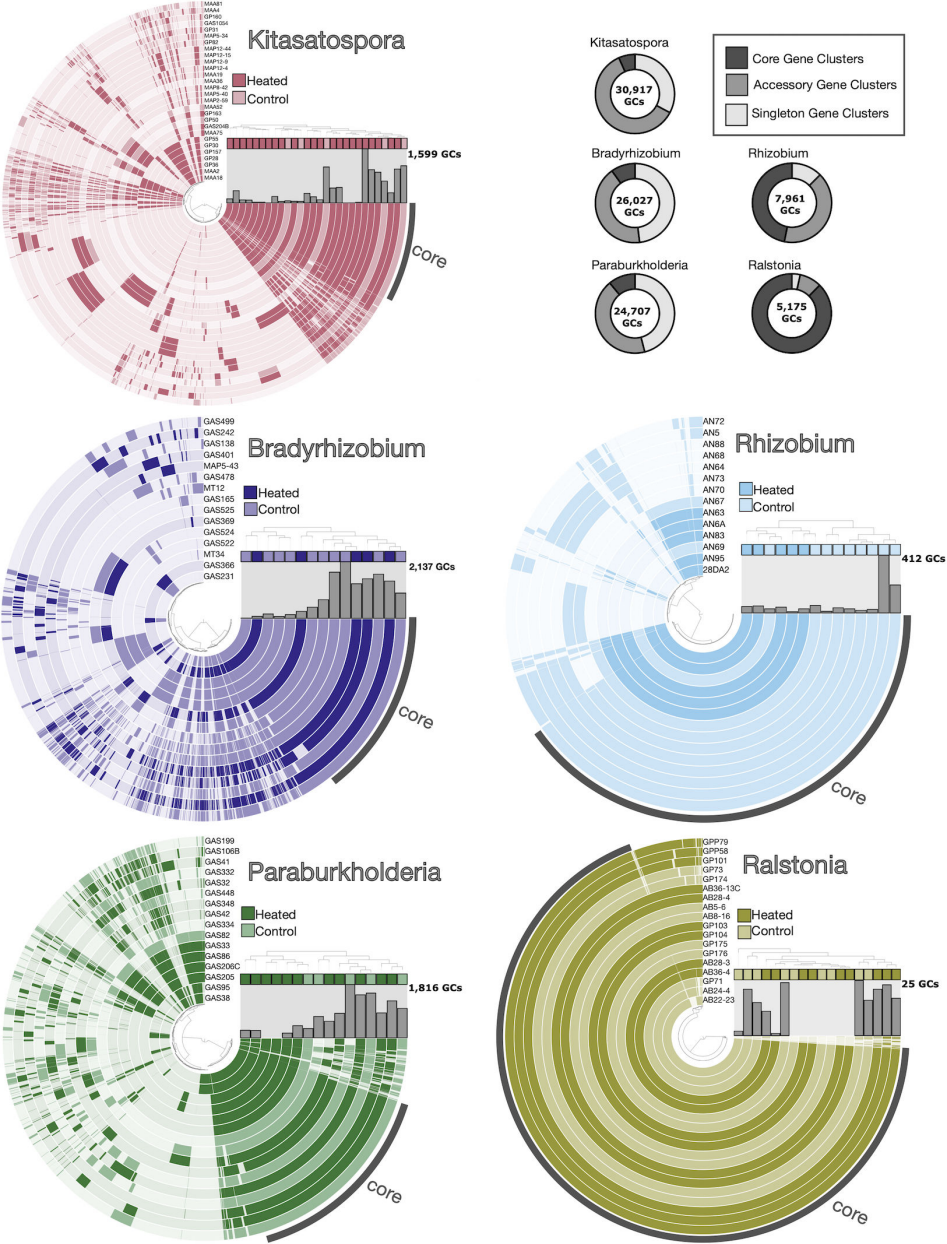
Pangenome composition and structure across clades. For each pangenome plot, concentric circles show gene cluster (GC) presence/absence across all genomes. The internal dendrogram orders genomes by gene cluster frequencies. For visualization, singletons were removed before plotting, and core GCs were labeled on each pangenome. Pangenomes are colored by clade, and genomes are colored by warming experiment treatment as shown in the legend. Bar graphs show the number of singleton GCs present in each genome. Finally, donut plots in the upper right panel illustrate pangenome structure or the portion of GCs belonging to the core, accessory, and singleton gene pools for each pangenome. See Table 1 for exact values.

We used comparative pangenomics to determine if genomes from heated treatments harbored different patterns of functional gene content compared to control treatments and whether these differences were consistent between clades. Pangenome structure and composition varied across phyla and between clades, ranging from more open to more closed pangenomes (Fig. 2; Table 1). For instance, the Betaproteobacteria *Ralstonia* pangenome contained relatively few GCs (*n* = 5,175 GCs) with nearly all of these belonging to the conserved core genome (*n* = 4,518 GCs) and very few strain-specific or singleton GCs. Conversely, Betaproteobacteria *Paraburkholderia* showed considerably higher gene richness with approximately five times more GCs (*n* = 24,707) with only 11% of GCs (*n* = 2,719) belonging to the core gene pool, and many singletons. We observed a contrasting pattern between the more open pangenome of Alphaproteobacteria *Bradyrhizobium* and the more closed pangenome of Alphaproteobacteria *Rhizobium*. We also observed a high portion of accessory and singleton GCs in the *Kitasatospora* pangenome, which is unsurprising as Actinobacteria are well known for their genomic and ecological diversity (79).

Patterns of gene variation across lineages evolve through various mechanisms that depend on both population parameters and ecological interactions (83, 84), and often these mechanisms are impossible to disentangle (85). Pangenomes are constantly in flux and are themselves shaped by environmental dynamics and ecological-evolutionary feedbacks (86). For example, lineages with large accessory gene pools have a deeper reserve of genes that may be shared within a population during times of stress or environmental change, facilitating ecological adaptation (87). However, a recent pangenome study found that environmental stressors including acidity, heat, drought, and salt stress all led to reductions in gene richness of *Bradyrhizobium diazoefficiens* (88), with accessory genes more prone to habitat-specific loss. These dynamics may reflect a transition between niche generalists with more open pangenomes versus niche specialists with more closed pangenomes (89).

We did not observe individual GCs strongly associating with heated or control treatments (*Q*-value < 0.1) for any of the clades with the exception of *Rhizobium* spp. (Fig. 2). In other words, there are no discrete collections of “heated core” or “control core” GCs in our pangenome collection. Because we did not see a strong treatment-specific signal at the GC level, we also investigated differences in functional gene content between treatments to discover annotations that were over- or underrepresented in genomes derived from chronically heated soils. Genomes were annotated hierarchically: GC annotations varied by functional orthologs (*n* = 334 KOfams; Table S4) or by functional units of genes within metabolic pathways (*n* = 35 KEGG pathway modules; Table S5). Finally, the estimated metabolic capabilities of strains also varied between treatments (*n* = 15 metabolic pathways; Table S6).

We found a few examples of functional annotations with strong support for differential associations between heated and control genomes. *Kitasatospora* genomes from heated soils were enriched in two metabolic pathways, the aerobic oxidation of oxaloacetate in the citrate cycle and the Arnon-Buchanan reductive citrate cycle (*Q*-value = 0.1) (Table S6). This suggests that central metabolism and energy production are important functional traits for *Kitasatospora* and in chronically warmed soils. *Rhizobium* genomes from control treatments were enriched (*Q*-value = 0.4) in functional orthologs related to amino acid and purine degradation and transcriptional regulation, while genomes from heated treatments were relatively enriched in functional orthologs involved in various nitrogen, carbon, and DNA-related metabolic processes (Table S4).

Following correction for multiple comparisons, nearly all of the functional annotations associated with heated or control genomes lack robust statistical support and may represent false positives (Tables S4 to S6). It is possible that chronic warming does not alter functional gene potential. Alternatively, due to the inherent complexity of natural microbial communities, we may have sampled too few individuals. This is clearly a limitation of this study, but we believe that a discussion of trends remains valuable. We summarize these observations below (uncorrected *P*-value < 0.05) while also acknowledging the need for additional data to verify these results.

GCs with functional orthologs related to carbon, nitrogen, DNA metabolism, gene regulation, and defense and stress mechanisms were differentially associated with heated and control genomes (uncorrected *P*-value < 0.05) (Table S4). However, these patterns differed across individual clades. For control genomes, this included relative enrichment of fatty acid, methane, and acetyl-CoA pathways for *Kitasatospora*; amino acid metabolism (polyamine biosynthesis) for *Bradyrhizobium*; purine degradation, nitrate assimilation, and amino acid degradation for *Rhizobium*; and central carbohydrate metabolism for *Paraburkholderia* (uncorrected *P*-value < 0.05) (Tables S5 and S6). Conversely, genomes from heated plots harbored pathways involved in central carbohydrate metabolism and amino acid (cysteine) biosynthesis for *Kitasatospora*; central metabolism and the gamma-aminobutyrate (GABA) shunt for *Bradyrhizobium*; and methionine and organic compound (phthalate) degradation for *Rhizobium* (uncorrected *P*-value < 0.05) (Tables S5 and S6). Since many of these functional annotations are within central metabolic processes, differences in metabolism pathways between heated and control groups likely reflect ecological-evolutionary feedback mechanisms shaping genome evolution.

Pathways associated with natural product biosynthesis and competition varied across warming treatments for the Proteobacteria clades. *Rhizobium* and *Bradyrhizobium* genomes from heated plots were relatively enriched in pathways related to beta-lactam biosynthesis and resistance, respectively, while *Paraburkholderia* genomes from control plots were enriched in the vancomycin resistance pathway (uncorrected *P*-value < 0.05) (Tables S5 and S6). For *Kitasatospora*, fosfomycin and betacyanin biosynthetic pathways were enriched in genomes from control plots, while calicheamicin and dihydrokalafungin biosynthetic pathways were enriched in genomes from heated plots (uncorrected *P*-value < 0.05) (Table S5). Differences in secondary metabolite repertoires could indicate different biotic competition pressures due to warming stress, or an indirect effect of the severe soil organic carbon depletion in the chronically heated soils.

### Carbohydrate-active enzymes

Given that differences in available carbon are the major environmental determinant driving ecological differentiation between heated and control soils, next we identified genes functionally annotated as carbohydrate-active enzymes (90), or CAZymes. These enzymes catalyze the breakdown, degradation, or modification of carbohydrates and glycoconjugates. The total number of CAZyme annotations did not differ across warming treatment for all clades (Table S2). Clade membership, but not warming treatment or the interaction between treatment and clade, was a strong predictor for CAZyme composition (PERMANOVA, *P*-value = 0.001, *R*^2^ = 0.85) (Fig. S2). Within clades, treatment is a significant predictor of CAZyme composition for *Rhizobium* (PERMANOVA, *P*-value = 0.019, *R*^2^ = 0.27), but not for the other clades.

While warming treatment did not explain variation in the total number or composition of CAZyme annotations across genomes, we observed individual CAZyme classes with different associations across heated and control genomes (uncorrected *P*-value < 0.05) (Table S7). For example, glycosyltransferase enzymes were relatively depleted in heated *Paraburkholderia* genomes. Heated genomes of *Rhizobium* and *Kitasatospora* were relatively enriched in polysaccharide lyase enzymes involved in the breakdown of alginate (uncorrected *P*-value < 0.05) (Table S7). Conversely, glycoside hydrolase enzymes with complex substrates such as xylan, chitin, and glycogen were relatively depleted in heated *Kitasatospora* genomes. Chronic soil warming depleted total soil organic matter and altered carbon composition, resulting in lower quantity as well as quality of carbon substrates in the heated versus control plots (20). Further, a previous study found that a greater portion of bacterial strains isolated from heated plots utilized complex carbon substrates (including xylan and cellulose) compared to strains isolated from control plots (26). Taken together, these data suggest that microbial substrate availability and carbon utilization shape microbial responses to soil warming.

### Codon usage bias (CUB)

CUB is an estimate of the degree to which an organism demonstrates non-random, or preferential, use of synonymous codons in genes. Codon bias relates to microbial growth and physiology through mechanisms involving protein translation accuracy and/or efficiency (91–93). We calculated CUB distributions between heated and control strains and across core and accessory genes, which may reflect differing selection pressures across gene pools or differences in horizontal gene exchange dynamics (94).

Different measurements of CUB offer various insights into codon usage across genes and gene expression levels across genomes (11). We measured CUB as the effective number of codons (ENC) Wright (68) and as the Measurement Independent of Length and Composition (MILC) (69), and CUB varied between heated and control genomes for both metrics. ENC is a standard metric indicating the number of codons in a gene, while MILC is a distance metric for a gene against a defined reference set. By examining both metrics, it’s possible to gain different perspectives on potential growth efficiency (ENC) and reflecting likely gene expression profiles (MILC).

ENC calculates the frequencies of different codons in a gene sequence and ranges from 20 (extreme bias, only a single codon is used for each amino acid) to 61 (no bias, all synonymous codons are equally used). Genome-wide gene-level ENC distributions differed between heated and control genomes for all clades (Wilcox rank sum test, *P*-value < 0.0001) except *Paraburkholderia* spp. and (*P*-value = 0.57) *Ralstonia* spp. (*P*-value = 0.73) (Fig. S3A). Core (Fig. S4A) and accessory (Fig. S5A) gene ENC distributions differed between heated and control genomes for all groups (*P*-value < 0.01) except *Ralstonia* (*P*-value > 0.6).

MILC calculates codon usage of a sequence as the distance against the expected distribution of a reference gene set. When determined against a set of highly expressed genes, like ribosomal proteins, higher values indicate more dissimilar patterns of codon usage. Genome-wide gene-level MILC distributions differed between heated and control genomes for all clades (Wilcox rank sum test, *P*-value < 0.001) except *Rhizobium* spp. (*P*-value = 0.18) (Fig. S3B). Core gene MILC distributions differed between heated and control genomes for all clades (P value < 0.03) besides *Bradyrhizobium* (*P*-value = 0.40) (Fig. S4B). Accessory gene MILC distributions differed between heated and control genomes for all clades (*P*-value < 0.01) except *Bradyrhizobium* spp. and *Rhizobium* (*P*-value > 0.1) (Fig. S5B).

**Fig 3:**
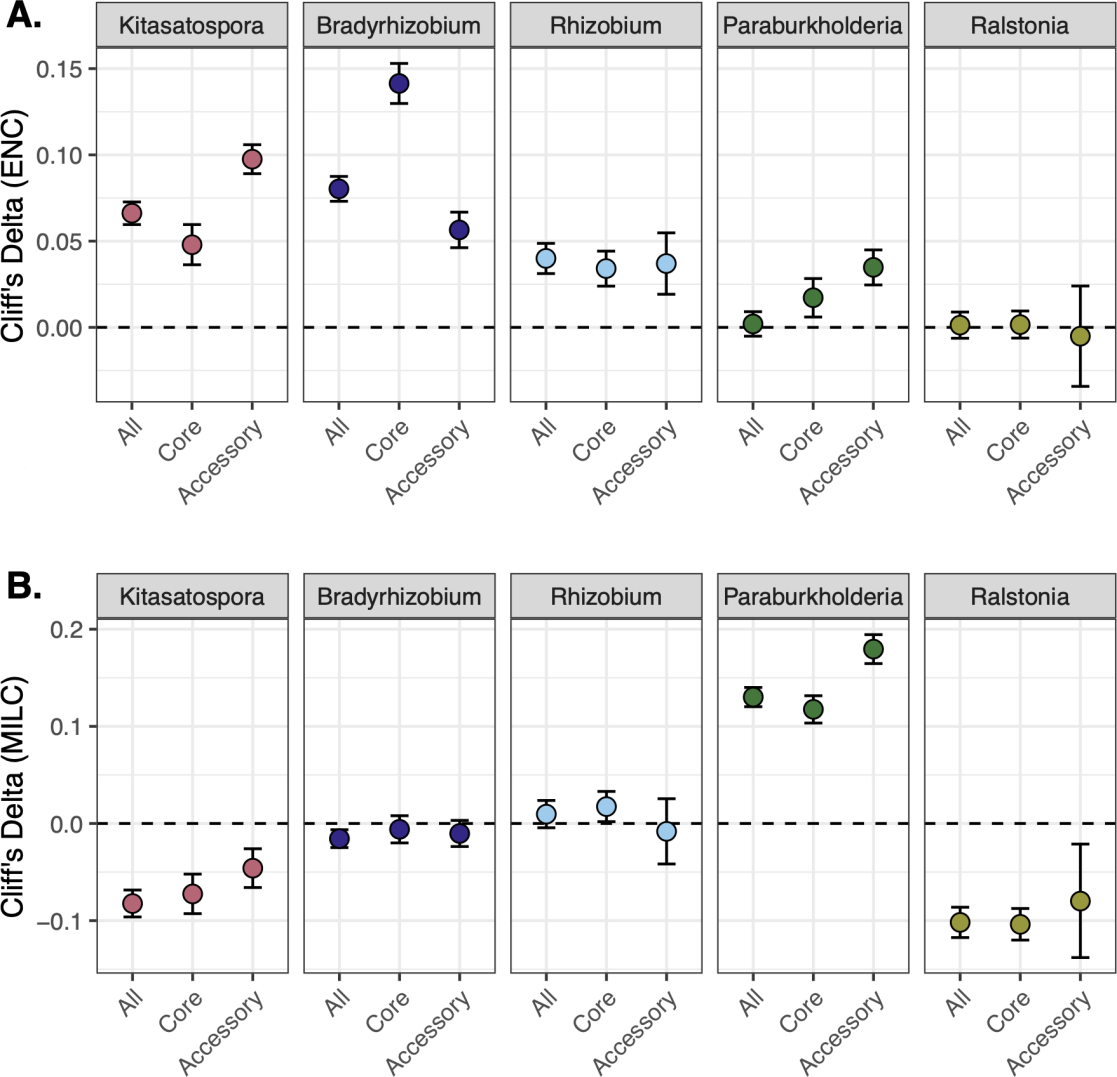
CUB effect size between heated and control. Plots illustrate Cliff’s delta, or the effect size, of global codon bias measurements between heated and control genomes for each pangenome clade and across all genes (All), core genes (Core), and accessory genes (Accessory). Cliff’s delta ranges from −1 to +1, with zero (dashed line) indicating full overlap between heated and control distributions. Positive values mean heated genomes have larger CUB distributions compared to control, and negative values mean control genomes have larger CUB distributions compared to heated. Circles show the Cliff’s delta estimate, and error bars indicate 95% confidence intervals. Core genes belong to gene clusters that are present in all genomes within a pangenome clade. Accessory genes belong to gene clusters that are shared by at least two, but not all genomes within a pangenome clade. Panel A shows Cliff’s delta for ENC (68) values, and Panel B shows Cliff’s delta for MILC (69) values. We calculated MILC values against a set of conserved ribosomal protein genes.

While gene-level CUB metrics show genome-wide distribution patterns, we also calculated Cliff’s delta (a non-parametric effect size estimate) to illustrate the magnitude of differences between heated and control genomes and across gene pools (Fig. 3). Here, more positive values indicate that codon bias values in heated genomes are greater than in control genomes, and more negative values indicate that codon bias values in control genomes are greater than heated. With a few exceptions, the effect size between heated and control genes is close to zero, indicating negligible differences between groups. Values greater than |d| 0.15 suggest a small effect size (95). We observe small, but meaningful, differences in core gene ENC distributions for *Bradyrhizobium* and MILC distributions for all gene pools in *Paraburkholderia* (Fig. 3).

Codon usage summarized for each genome facilitates comparing between treatment and gene pools. Median ENC and MILC varied significantly across gene pools for all clades (ANOVA, *P*-value < 0.001) (Fig. S6). Strain-level median ENC codon usage differed between heated and control *Kitasatospora* genomes for ”All” and ”Accessory” gene pools (Wilcox rank sum test, *P*-value ≤ 0.05) (Fig. S6A). Strain-level MILC codon usage nearly differed between heated and control *Paraburkholderia* genomes for core and accessory gene pools (*P*-value ≤ 0.1) (Fig. S6B). Long-term warming was associated with less ENC codon bias (higher values) for Actinobacteria and Alphaproteobacteria clades but not Betaproteobacteria clades (Fig. 3A; Fig. S6A). High MILC values, or codon usage patterns less similar to highly expressed ribosomal protein coding genes, were observed in *Paraburkholderia* heated genomes compared to control genomes (Fig. 3B; Fig. S6B). For *Paraburkholderia*, this suggests that genomes from chronically heated soils have different global protein expression profiles (i.e., potentially related to maximum growth rate). For all clades, accessory genes have distinct codon bias distributions compared to core genes, which may contribute to traits related to growth rate and efficiency.

Genomic traits like ribosomal operon copy number often serve as proxies for traits that are more challenging to measure, like growth rate or carbon use efficiency. We asked if there was a relationship between global codon bias and 16S rRNA gene copy number, since both traits are ultimately connected to growth and physiology. It is well documented that small subunit ribosomal (SSU) rRNA copy number reflects microbial growth traits and ecological strategies (96), with more ribosomal operon copies enabling faster doubling times. Finally, maximum growth rate was shown to be inversely correlated with carbon use efficiency (97), further linking these traits. These relationships are so robust that there are tools available to infer growth rate and efficiency based on ribosomal copy number and codon bias (98, 99), though they are unable to make accurate predictions for slower-growing organisms.

**Fig 4:**
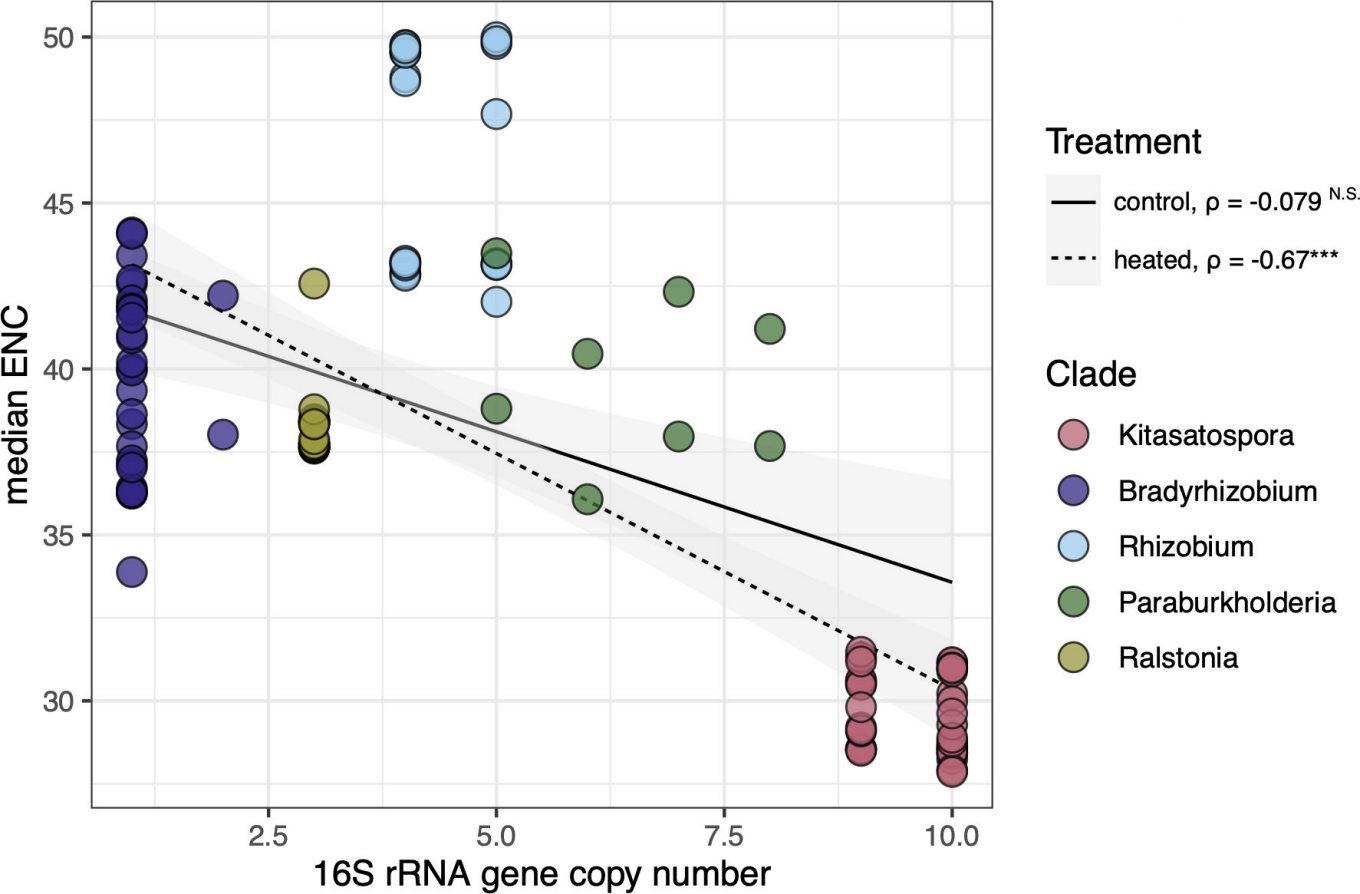
CUB versus 16S rRNA gene copy number. Plot illustrates the relationships between global codon usage bias (*y*-axis) and 16S rRNA gene copy number (*x*-axis) (Table S2). We omitted genome assemblies with over 20 contigs (see Harvard Forest soil warming experiment bacterial genome collection section). Circles show median values of strain-level ENC codon usage for each genome across core and accessory gene pools and are colored according to pangenome clade. Linear regression is plotted for heated (dashed line) and control (solid line) treatment. Shaded areas depict 95% confidence intervals. Spearman’s *ρ* is reported, and asterisks note *P*-value < 0.0001.

In general, genomes with more 16S rRNA gene copies have stronger CUB (ENC) (Fig. 4). We observed a significant negative relationship between median ENC codon bias and 16S rRNA gene copy number for heated (Spearman’s ρ = −0.67, *P*-value < 0.0001) but not control genomes (Spearman’s ρ −0.079, *P*-value = 0.53). This relationship differs between treatment (slope of linear model differs between groups, *P*-value = 0.048) (Fig. 4). Within clades, SSU copy number does not vary between heated and control genomes, suggesting that these organisms experience different selective pressures on growth efficiency.

Growing evidence suggests environmental drivers of microbial community-level codon usage. For instance, a recent cross-biome metagenomic study demonstrated the direct impact of microbial habitat on codon bias and amino acid usage after controlling for other non-environmental variables (100). Ultimately, a number of factors influence codon usage patterns in microbial genomes, including genome-wide G + C content (101) as well as tRNA abundances Ikemura (102, 103). Global CUB correlates with microbial life history strategies, growth rate, and ecological adaptations (104–106). These studies support the possibility that adaptations related to CUB may result in trade-offs between resource acquisition and growth. Traits that appear to emerge in soil microbiomes exposed to long-term warming (i.e., growth and substrate utilization [24, 26]) could lead to a decoupling of the relationship between 16S rRNA gene copy number and global CUB.

### Conclusion

We examined genomes of bacteria exposed to decades of climate warming to understand whether microbes evolve and adapt to warmer soils. Over three decades, heated soils have lost 34% of soil organic matter (21), accompanied by greater depletion of carbohydrates and other relatively more accessible forms of heterotrophic substrates (20). Warming caused a reduction in fungal but not bacterial biomass (22, 24), suggesting a seasonal shift toward a more bacterial-dominated ecosystem and local adaptation to enable this change. Using comparative pangenome approaches, we identified genomic traits related to growth efficiency (Fig. 3 and 4) and resource acquisition (Fig. S2; Tables S4 to S7), which supports our previous observations that depleted soil organic matter in warming plots leads to more oligotrophic life history strategies (24) and utilization of more complex carbon substrates (26).

Microbial populations tend to adapt to local environmental conditions (107). However, evidence for ecological adaptation (or lack thereof) varies across environmental, spatial, and phylogenetic distances (108–111). The question here is, does local adaptation act along the environmental and temporal scales of the Harvard Forest warming experiment? Based on an average generation time of 2 weeks for soil microbial communities estimated using 18O-labeled water (38), 30 years corresponds to a minimum of 500 generations in our Harvard Forest system. Other studies have observed adaptation to temperature stress across similar generations, although these are predominantly under highly controlled experimental conditions.

In laboratory settings, microbes adapt to increasing temperatures rapidly (112, 113). In a previous study, after 1,500 generations of selective temperature stress, the fungus *Neurospora* adapted to increased temperature through life history trait trade-offs, including increased resource allocation to spores but decreased growth rate and higher respiration (114). Insights into microbial responses to climate change across longer time scales that impact ecosystem-level functions are narrow due to limited access to long-term warming experiments. Even more rare are observations of local adaptation *in situ*. A small but growing body of research demonstrates ecological and evolutionary responses resulting in adaptation to environmental change on similar timescales (32, 115). For example, in a recent reciprocal transplant study across a natural temperature gradient, ecological and evolutionary feedbacks were detected on a timescale of 1.5 years (116). In this analysis, consistent mutations were detected in genes related to nutrient acquisition and stress responses.

Microbial culture collections are invaluable resources for linking organism-level responses to climate change and ecosystem function. However, building and maintaining culture collections are labor-intensive and costly. In this study, we observed compelling trends in genomic traits related to growth resource acquisition (i.e., functional gene content) and life history strategies (i.e., CUB), but we admittedly lack strong statistical support. Soil systems are inherently complex. Disentangling ecological and evolutionary processes shaping soil microbial dynamics and identifying adaptive traits requires either a very strong signal or many representative genomes. Yet ecologically explicit culture collections offer unique opportunities to bridge sequence data with function (117).

The pangenomes of five clades (Fig. 2) reflect organisms with different growth and nutrient acquisition strategies, based on carbohydrate-activated enzymes, functional gene content, and CUB. Our comparative pangenome data supports our previous works suggesting the importance of microbial traits related to growth efficiency and carbon substrate utilization (26, 41). Trends in the functional gene potential of genomes from heated plots suggest increased competition under a regime of more limited resources due to chronic warming. Ultimately, lineage-specific adaptations to climate change may aggregate at the community level, resulting in altered soil biogeochemical functions (118). Future studies investigating the physiology of these isolates, focusing on the potential trade-offs between growth efficiency and resource utilization, will further strengthen our ability to predict microbial function from genome sequences. Quantifying ecological-evolutionary responses to climate change across temporal and environmental scales will support our ability to better predict ecosystem function in a changing world.

## Data Availability

All genomic data used in this study are publicly available. Genome sequence dataare archived on the SRA under BioProjects PRJNA944970, PRJNA944974, PRJNA944975, PRJNA944977, and PRJNA944978 with BioSample accession numbers also referenced in Table S1. All draft genome assemblies are also archived on the DOE JGI’s Integrated Microbial Genomes and Microbiomes (IMG/M) under study names “Sequencing of bacterial isolates from soil warming experiment in Harvard Forest, Massachusetts, USA,” and “Using genomics to understand microbial adaptation to soil warming” with GOLD Study IDs Gs0121248 and Gs0156716. IMG Taxon/Genome ID for each assembly is referenced in Table S1.

## SUPPLEMENTAL MATERIAL

Supplemental material msphere.00059-25-s0001.docx Supplemental figures and captions for supplemental tables.

Supplemental tables msphere.00059-25-s0002.pdf Tables S1 to S7.

ASM does not own the copyrights to Supplemental Material that may be linked to, or accessed through, an article. The authors have granted ASM a non-exclusive, world-wide license to publish the Supplemental Material files. Please contact the corresponding author directly for reuse.
