# Supplementary material for: Pangenomes suggest ecological-evolutionary responses to experimental soil warming
Source: mSphere. 2025 Mar 19;10(4):e00059-25. doi: 10.1128/msphere.00059-25 (PMC12039271; doi:10.1128/msphere.00059-25)
Supplement: Supplemental material — Supplemental figures and captions for supplemental tables. [file msphere.00059-25-s0001.docx]

**Supplementary Information**

**Figure S1. 16S rRNA gene phylogeny**. Tree shows the phylogenetic relationships between all 91 isolates (Table S1) constructed from 16S rRNA gene nucleotide sequences derived from anvi'o HMM searches. Genome names are colored according to treatment group, with heated in red and control in black. Phylogenetic clades are also labeled and colored according to their pangenome group. Scale bar indicates nucleotide substitutions per site. Bootstrap values greater than 70 from 100 iterations are noted on tree branches. Tree is mid-rooted. Note, *Paraburkholderia* genomes GAS33, GAS82, GAS205, and GAS206C did not have 16S rRNA gene hits, so these strains are omitted from the tree. Circles indicate 16S rRNA gene sequences with > 99% identity to partial 16S rRNA gene sequences (254 bp) from dominant subset community (n=155 OTUs, rank abundance) from a previous amplicon study conducted at the Harvard Forest warming experiment (DeAngelis *et al.*, 2015). Alphaproteobacteria taxa NewOTU354050 and 999357 had greater relative abundances in heated communities, while Betaproteobacteria taxon 1105280 had greater relative abundances in control communities. None of these OTUs were indicator taxa.

**Figure S2. CAZyme composition across clades.** Plot shows non-metric multidimensional scaling (NMDS) ordination of CAZyme composition (Bray-Curtis distances) across all genomes. Each point represents the collection of carbohydrate-active enzymes annotated with the dbCAN HMM database (HMMdb release 11.0) (Zhang *et al*., 2018; Zheng *et al.*, 2023) for each genome. Points are colored by clade and shapes correspond to warming experiment treatment according to legend. Clade membership, but not treatment or the interaction between treatment and clade, was a strong predictor for CAZyme composition (PERMANOVA, P-value=0.001, R^2^=0.85).


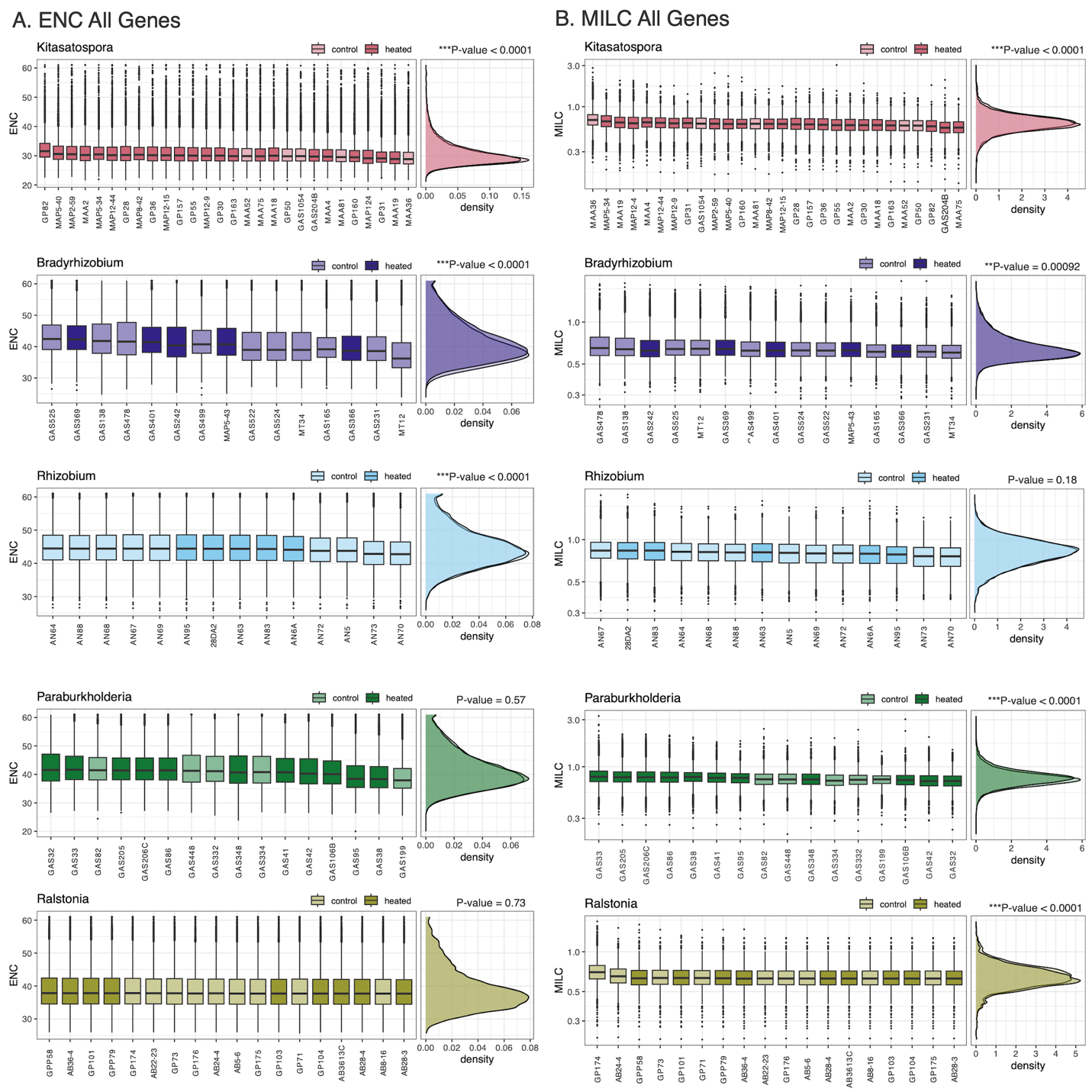


**Figure S3. Gene-level genome-wide codon usage distributions.** Panels illustrate global gene-level codon usage distributions for all strains across clades. Each boxplot shows the interquartile range of codon usage values for all genes, and the line is the median, whiskers are 1.5 interquartile range, and black points are outliers. Boxplots are colored by clade and treatment. Plots to the right show the codon usage density distributions by treatment. Figure S3A shows ENC (Wright, 1990) distributions, and Figure S3B shows MILC (Supek & Vlahoviček, 2005) distributions. We calculated MILC values against a set of conserved ribosomal protein genes. Wilcox rank sum test P-values for differences in codon usage between warming treatment are reported.


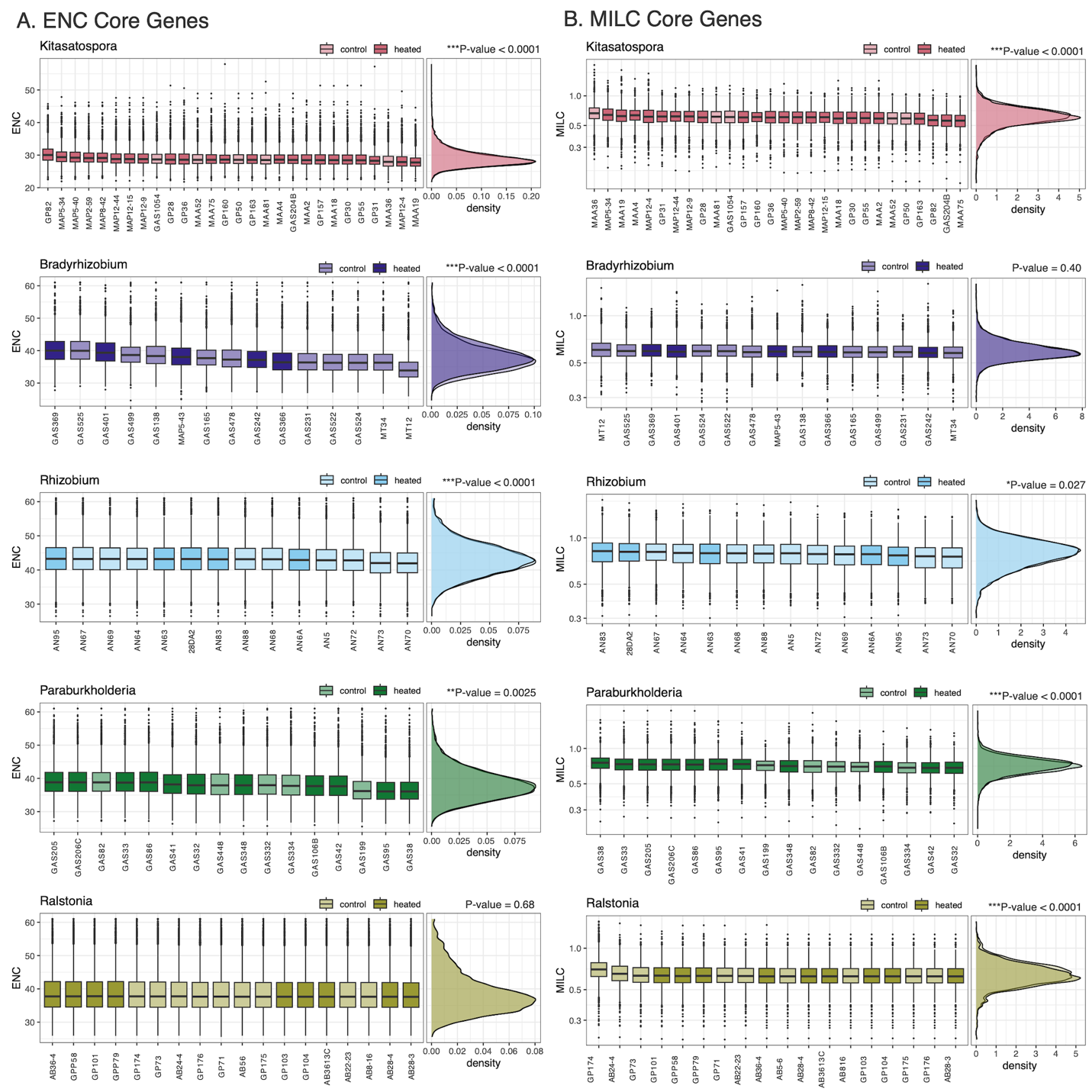


**Figure S4. Gene-level core genome codon usage distributions.** Panels illustrate gene-level core genome codon usage distributions for all strains across all clades. Each boxplot shows the interquartile range of codon usage values for core genes, and the line is the median, whiskers are 1.5 interquartile range, and black points are outliers. Boxplots are colored by clade and treatment. Plots to the right show the codon usage density distributions by treatment. Figure S4A shows ENC (Wright, 1990) distributions, and Figure S4B shows MILC (Supek & Vlahoviček, 2005) distributions. We calculated MILC values against a set of conserved ribosomal protein genes. Wilcox rank sum test P-values for differences in codon usage between warming treatment are reported.


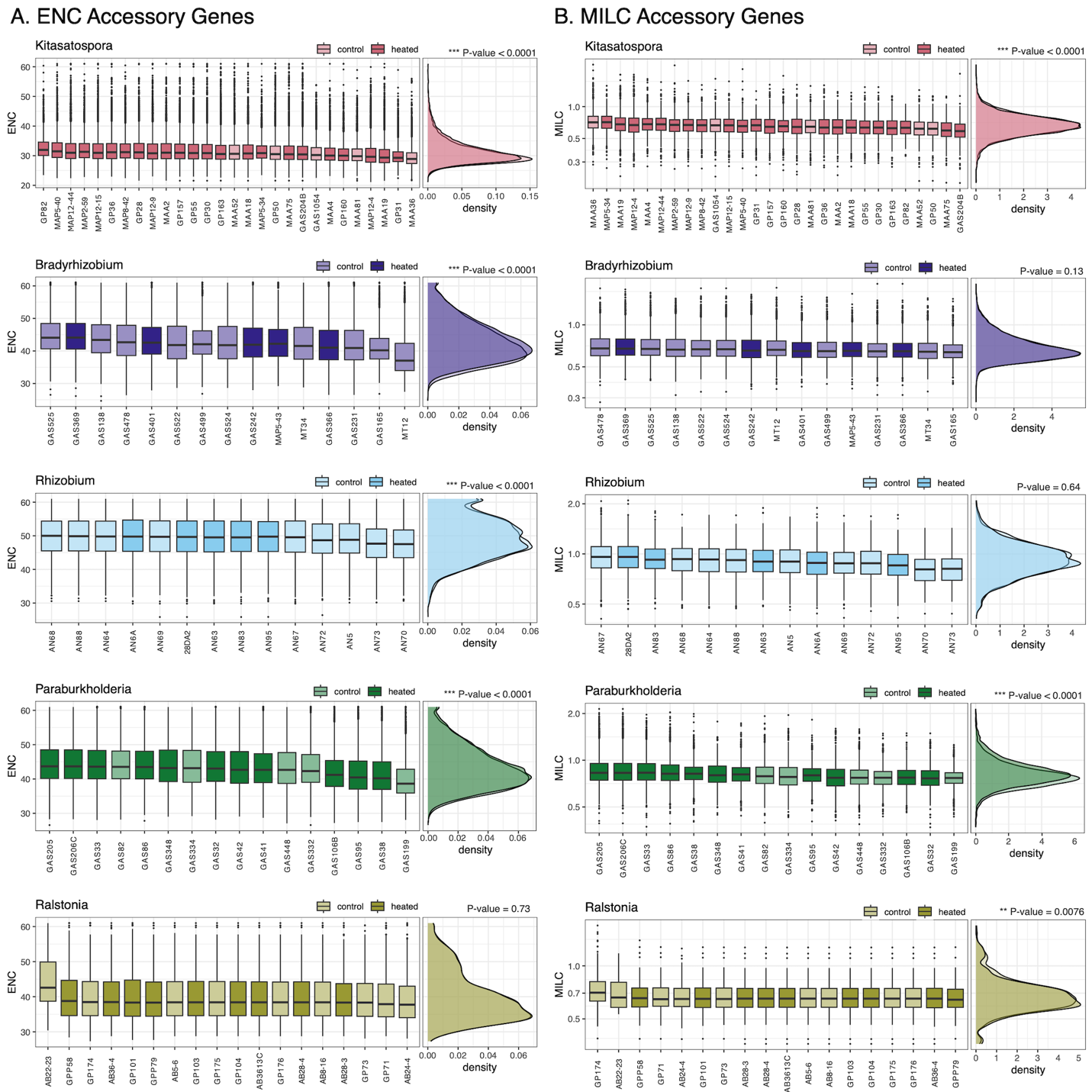


**Figure S5. Gene-level accessory genome codon usage distributions.** Panels illustrate the gene-level accessory genome codon usage distributions for all strains across all clades. Note, singletons genes were not included in the accessory gene pool for this analysis. Each boxplot shows the interquartile range of codon usage values for accessory genes, and the line is the median, whiskers are 1.5 interquartile range, and black points are outliers. Boxplots are colored by clade and treatment. Plots to the right show the codon usage density distributions by treatment. Figure S5A shows ENC (Wright, 1990) distributions, and Figure S5B shows MILC (Supek & Vlahoviček, 2005) distributions. We calculated MILC values against a set of conserved ribosomal protein genes. Wilcox rank sum test P-values for differences in codon usage between warming treatment are reported.


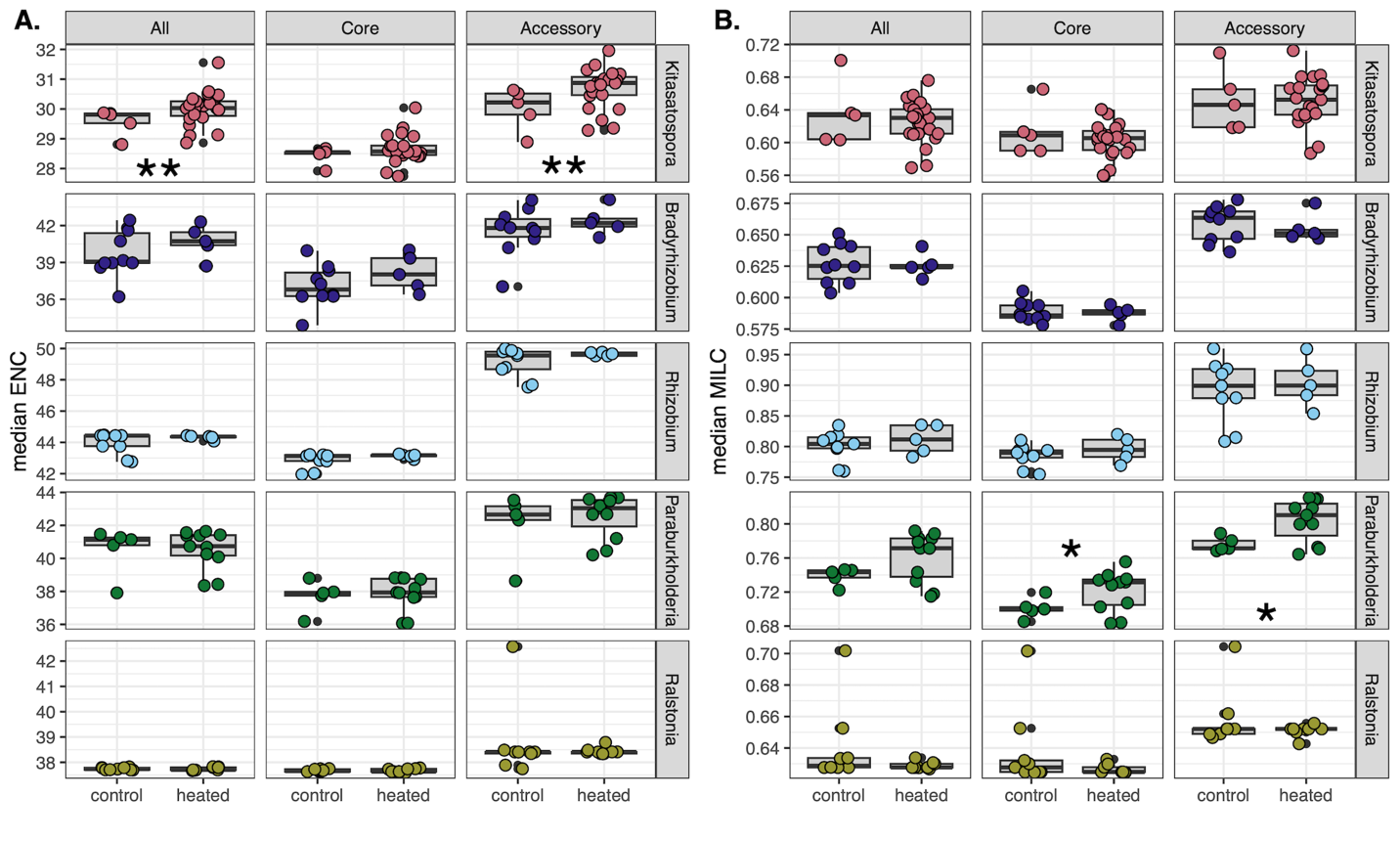


**Figure S6. Strain-level median CUB values across gene pools.** Plots illustrate the strain-level (i.e., summarized per genome) median codon usage between treatment groups across clades and across gene pools. Columns are codon usage summary for all genes (All), core genes (Core), or accessory genes (Accessory). Core genes belong to gene clusters that are present in all genomes within a pangenome clade. Accessory genes belong to gene clusters that are shared by at least two, but not all genomes within a pangenome clade. Note, singleton genes were not included in the accessory gene pool for this analysis. Boxplots show the interquartile range of codon usage values for control and heated genomes, and the line is the median, whiskers are 1.5 interquartile range, and black points are outliers. Circles show median codon usage values for each strain and are colored by clade. Figure 4A shows ENC (Wright 1990) values, and Figure 4B shows MILC (Supek *et al.*, 2005) values. We calculated MILC values against a set of conserved ribosomal protein genes. Asterisks indicate Wilcox rank sum test P-values for differences in median codon usage between treatment ≤ 0.05 (*); < 0.10 > 0.05 (**).

**Table S1. Isolation metadata for bacterial isolates.** The 91 isolates in this study include *Kitasatospora* spp., *Bradyrhizobium* spp., *Paraburkholderia* spp., and *Rhizobia* spp. clades. Columns report isolate ID, phylum, organism name, warming experiment treatment, soil horizon (na values are unknown), original isolation media, year isolated, genome sequencing platform, sequencing facility, NCBI BioSample accession number, and IMG taxon ID of the genome assembly. Media abbreviations include: VL55 amended with xylan, plant ploymers (PP), or readily oxidized carbon (ROC); humic acid vitamin agar (HVA); lignin soybean flour vitamin agar (LSFA); modified carboxymethyl cellulose (CMC); International *Streptomyces* Project-2 Medium (ISP2); and 1% glucose/0.4% potato infusion (1% glu). See references within (Pold *et al.*, 2016; Domeignoz-Horta *et al.*, 2020) for media formulations and additional isolation conditions. Note the 20 genomes in the sequencing facility column that were previously sequenced, indicated with asterisks. All other genomes were generated for this study.

**Table S2. Genome metadata for draft genome assemblies.** Columns report genome ID, pangenome clade, warming experiment treatment, number of contigs in the assembly, total assembly length, percent G+C, percent estimated genome completeness and redundancy (plus estimated confidence), total number of genes, average gene length, number of genes per kb, number of unique or singleton gene clusters, total number of gene clusters, 16S rRNA gene number, and the total number of carbohydrate-active enzyme (i.e., CAZymes) annotations. We used the program anvi-estimate-genome-completeness to estimate genome completion and redundancy based on presence of single-copy genes. We used the program anvi-run-hmms to determine 16S rRNA gene copy numbers with Hidden Markov Model (HMM) searches to anvi'o HMM sources Ribosomal_RNA_16S. We used anvi-run-cazymes to run HMM searches against dbCAN HMMdb v11 (Zhang *et al*., 2018; Zheng *et al.*, 2023).

**Table S3. ONT minION assembly metadata and quality control metrics.** For all draft genomes sequenced and assembled in house using ONT minION, columns report isolate ID, phylum, pangenome clade, *de novo* or hybrid assembly (see Methods), assembly coverage (X), taxonomic classification determined by CheckM (Parks *et al.*, 2015), assembly length, percent G+C, number of contigs, N50, and completeness and contamination as estimated by CheckM.

**Table S4. Functional enrichment metrics for KOfam annotated gene clusters.** Columns are KOfam descriptions, enrichment score (see Methods and Shaiber *et al*., 2020), unadjusted P-values, Q-values adjusting for multiple tests, associated warming experiment treatment, KO identifier, corresponding gene clusters, the portion of heated (p_Heated) and control (p_Control) genomes that harbor each functional annotation, and clade. Note, only functional annotations with unadjusted P-values < 0.05 are reported.

**Table S5. Functional enrichment metrics for KEGG annotated gene clusters.** Columns are KEGG module descriptions, enrichment score (see Methods and Shaiber *et al*., 2020), unadjusted P-values, Q-values adjusting for multiple tests, associated warming experiment treatment, KEGG identifier, corresponding gene clusters, the portion of heated (p_Heated) and control (p_Control) genomes that harbor each functional annotation, and clade. Note, only functional annotations with unadjusted P-values < 0.05 are reported.

**Table S6. Functional enrichment metrics for complete metabolic modules.** Columns are KEGG complete metabolic module descriptions (Kanehisa *et al.*, 2012), enrichment score (see Methods and Shaiber *et al*., 2020), unadjusted P-values, Q-values adjusting for multiple tests, associated warming experiment treatment, KEGG metabolic module identifier, corresponding genomes, the portion of heated (p_Heated) and control (p_Control) genomes that harbor each metabolic module, and clade. Note, only metabolic modules with unadjusted P-values < 0.05 are reported.

**Table S7. Functional enrichment metrics for carbohydrate-active enzymes (CAZymes)**. Columns are CAZyme HMM name, enzyme class, family activity description and EC number, enzyme substrate, enrichment score (see Methods and Shaiber *et al*., 2020), unadjusted P-values, Q-values adjusting for multiple tests, associated warming experiment treatment, predicted substrate, corresponding gene clusters, the portion of control (p_Control) and heated (p_Heated) genomes that harbor each CAZyme annotation, and clade. Note, only CAZyme annotations with unadjusted P-values < 0.05 are reported. Enzyme descriptions were parsed from the Carbohydrate-Active enZYmes Database webpages of www.cazy.org.
