# Supplementary material for: Pangenomes suggest ecological-evolutionary responses to experimental soil warming
Source: mSphere. 2025 Mar 19;10(4):e00059-25. doi: 10.1128/msphere.00059-25 (PMC12039271; doi:10.1128/msphere.00059-25)
Supplement: Supplemental tables — Tables S1 to S7. [file msphere.00059-25-s0002.pdf]

Table S1. Isolate Metadata

Isolate	Phylum	Organism Name	Treatment	Horizon	Isolation Media	Isolation Date	Sequence Platform	Sequencing Facility	Biosample	IMG taxon ID
**Actinobacteria *Kitasatospora* spp. clade**										
GAS1054	Actinobacteria	Kitasatospora sp. GAS1054	control	mineral	VL55 PP	2014	ONT MinION	DeAngelis Lab	SAMN33769326	2958935987
GAS204B	Actinobacteria	Kitasatospora sp. GAS204B	heated	mineral	VL55 PP	2014	Illumina NovaSeq S4	DOE JGI	SAMN33769410	2981703317
GP28	Actinobacteria	Kitasatospora sp. GP28	heated	organic	oatmeal	2014	ONT MinION	DeAngelis Lab	SAMN33771170	2953957014
GP30	Actinobacteria	Kitasatospora sp. GP30	heated	organic	oatmeal	2014	Illumina NovaSeq S4	DOE JGI	SAMN33771171	2981846400
GP31	Actinobacteria	Kitasatospora sp. GP31	heated	organic	oatmeal	2014	ONT MinION	DeAngelis Lab	SAMN33771172	2955925447
GP36	Actinobacteria	Kitasatospora sp. GP36	heated	organic	oatmeal	2014	ONT MinION	DeAngelis Lab	SAMN33771173	2940444706
GP50	Actinobacteria	Kitasatospora sp. GP50	control	mineral	oatmeal	2014	ONT MinION	DeAngelis Lab	SAMN33771174	2953929336
GP55	Actinobacteria	Kitasatospora sp. GP55	heated	mineral	oatmeal	2014	PacBio RS, PacBio RS II	DOE JGI*	SAMN04515641	2617270923
GP82	Actinobacteria	Kitasatospora sp. GP82	heated	mineral	oatmeal	2014	Illumina NovaSeq S4	DOE JGI	SAMN33771176	2982040100
GP157	Actinobacteria	Kitasatospora sp. GP157	heated	organic	HVA	2014	ONT MinION	DeAngelis Lab	SAMN33769410	2958916086
GP160	Actinobacteria	Kitasatospora sp. GP160	heated	mineral	HVA	2014	ONT MinION	DeAngelis Lab	SAMN33771167	2958909016
GP163	Actinobacteria	Kitasatospora sp. GP163	heated	mineral	LSFV	2014	ONT MinION	DeAngelis Lab	SAMN33771168	2953937211
MAA2	Actinobacteria	Kitasatospora sp. MAA2	heated	organic	VL55 xylan	2020	ONT MinION	DeAngelis Lab	SAMN33771178	2953912599
MAA4	Actinobacteria	Kitasatospora sp. MAA4	heated	organic	VL55 xylan	2020	Illumina NovaSeq S4	DOE JGI	SAMN33771180	2982008629
MAA18	Actinobacteria	Streptomycetaceae bacterium MAA18	heated	mineral	VL55 xylan	2020	Illumina NovaSeq S4	DOE JGI	SAMN33771207	2981232351
MAA19	Actinobacteria	Kitasatospora sp. MAA19	heated	mineral	VL55 xylan	2020	Illumina NovaSeq S4	DOE JGI	SAMN33771177	2960241103
MAA36	Actinobacteria	Kitasatospora sp. MAA36	control	organic	VL55 xylan	2020	ONT MinION	DeAngelis Lab	SAMN33771179	2958928483
MAA52	Actinobacteria	Kitasatospora sp. MAA52	control	mineral	VL55 xylan	2020	ONT MinION	DeAngelis Lab	SAMN33771181	2940436776
MAA75	Actinobacteria	Kitasatospora sp. MAA75	heated	organic	ISP2	2020	ONT MinION	DeAngelis Lab	SAMN33771182	2953921507
MAA81	Actinobacteria	Kitasatospora sp. MAA81	control	organic	ISP2	2020	ONT MinION	DeAngelis Lab	SAMN33771183	2953904123
MAP2-59	Actinobacteria	Kitasatospora sp. MAP2-59	heated	mineral	VL55 ROC	2020	ONT MinION	DeAngelis Lab	SAMN33771188	2958901192
MAP5-34	Actinobacteria	Kitasatospora sp. MAP5-34	heated	mineral	ISP2	2020	Illumina NovaSeq S4	DOE JGI	SAMN33771189	2963575613
MAP5-40	Actinobacteria	Kitasatospora sp. MAP5-40	heated	mineral	ISP2	2020	ONT MinION	DeAngelis Lab	SAMN33771190	2953966083
MAP8-42	Actinobacteria	Kitasatospora sp. MAP8-42	heated	mineral	VL55 ROC	2020	ONT MinION	DeAngelis Lab	SAMN33771191	2955909675
MAP12-4	Actinobacteria	Kitasatospora sp. MAP12-4	heated	mineral	oatmeal	2020	ONT MinION	DeAngelis Lab	SAMN33771185	2939854634
MAP12-9	Actinobacteria	Kitasatospora sp. MAP12-9	heated	mineral	oatmeal	2020	Illumina NovaSeq S4	DOE JGI	SAMN33771187	2963582266
MAP12-15	Actinobacteria	Kitasatospora sp. MAP12-15	heated	mineral	oatmeal	2020	Illumina NovaSeq S4	DOE JGI	SAMN33771184	2982032058
MAP12-44	Actinobacteria	Kitasatospora sp. MAP12-44	heated	organic	oatmeal	2020	PacBio Sequel IIe	DOE JGI	SAMN33771186	2974460287
**Alphaproteobacteria *Bradyrhizobium* spp. clade**										
GAS138	Alphaproteobacteria	Bradyrhizobium erythrophlei GAS138	control	mineral	VL55 PP	2014	PacBio RS, PacBio RS II	DOE JGI*	SAMN05443248	2695421015
GAS165	Alphaproteobacteria	Bradyrhizobium lablabi GAS165	control	mineral	VL55 PP	2014	PacBio RS, PacBio RS II	DOE JGI*	SAMN05877939	2747843221
GAS231	Alphaproteobacteria	Afipia sp. GAS231	control	mineral	VL55 PP	2014	PacBio RS, PacBio RS II	DOE JGI*	SAMN05444050	2690315678
GAS242	Alphaproteobacteria	Bradyrhizobium erythrophlei GAS242	heated	mineral	VL55 PP	2014	PacBio RS, PacBio RS II	DOE JGI*	SAMN05444169	2695420948
GAS366	Alphaproteobacteria	Bradyrhizobium sp. GAS366	heated	mineral	VL55 PP	2014	ONT MinION	DeAngelis Lab	SAMN33771221	2929879271
GAS369	Alphaproteobacteria	Bradyrhizobium canariense GAS369	heated	mineral	VL55 PP	2014	PacBio RS, PacBio RS II	DOE JGI*	SAMN05444158	2693430033
GAS401	Alphaproteobacteria	Bradyrhizobium erythrophlei GAS401	heated	na	VL55 PP	2014	PacBio RS, PacBio RS II	DOE JGI*	SAMN05444170	2695420919
GAS478	Alphaproteobacteria	Bradyrhizobium erythrophlei GAS478	control	mineral	VL55 PP	2014	PacBio RS, PacBio RS II	DOE JGI*	SAMN05443247	2698537050
GAS499	Alphaproteobacteria	Bradyrhizobium lablabi GAS499	control	mineral	VL55 PP	2014	PacBio RS, PacBio RS II	DOE JGI*	SAMN05444159	2698536816
GAS522	Alphaproteobacteria	Bradyrhizobium lablabi GAS522	control	mineral	VL55 PP	2014	PacBio RS, PacBio RS II	DOE JGI*	SAMN05444171	2693429786
GAS524	Alphaproteobacteria	Bradyrhizobium ottawaense GAS524	control	mineral	VL55 PP	2014	PacBio RS, PacBio RS II	DOE JGI*	SAMN05444163	2693430034
GAS525	Alphaproteobacteria	Afipia broomeae GAS525	control	mineral	VL55 PP	2014	PacBio RS, PacBio RS II	DOE JGI*	SAMN05880572	2740892596
MAP5-43	Alphaproteobacteria	Bradyrhizobium sp. MAP5-43	heated	mineral	ISP2	2020	ONT MinION	DeAngelis Lab	SAMN33771222	2930805785
MT12	Alphaproteobacteria	Bradyrhizobium erythrophlei MT12	control	organic	VL55 xylan	2014	PacBio RS, PacBio RS II	DOE JGI*	SAMN05444164	2690316366
MT34	Alphaproteobacteria	Bradyrhizobium lablabi MT34	control	organic	VL55 xylan	2014	PacBio RS, PacBio RS II	DOE JGI*	SAMN05444321	2698536699

Table S1. Isolate Metadata

Alphaproteobacteria *Rhizobium* spp. clade

28DA2	Alphaproteobacteria	Rhizoboum sp. 28DA2	heated	subsurface	BioSep; Bandounas ligni	2013	ONT MinION	DeAngelis Lab	SAMN33771228	2929580477
AN5	Alphaproteobacteria	Rhizobium sp. AN5	control	surface	BioSep; Bandounas ligni	2013	PacBio RS, PacBio RS II	DOE JGI*	SAMN05216358	2617270923
AN63	Alphaproteobacteria	Rhizoboum sp. AN63	heated	subsurface	BioSep; Bandounas ligni	2013	ONT MinION	DeAngelis Lab	SAMN33771229	2929873235
AN64	Alphaproteobacteria	Rhizoboum sp. AN64	control	subsurface	BioSep; Bandounas ligni	2013	ONT MinION	DeAngelis Lab	SAMN33771230	2929597866
AN67	Alphaproteobacteria	Rhizoboum sp. AN67	control	surface	BioSep; Bandounas ligni	2013	ONT MinION	DeAngelis Lab	SAMN33771231	2930909959
AN68	Alphaproteobacteria	Rhizobium sp. AN68	control	surface	BioSep; Bandounas ligni	2013	ONT MinION	DeAngelis Lab	SAMN33771224	2935258951
AN69	Alphaproteobacteria	Rhizoboum sp. AN69	control	surface	BioSep; Bandounas ligni	2013	ONT MinION	DeAngelis Lab	SAMN33771232	2935252894
AN6A	Alphaproteobacteria	Rhizobium sp. AN6A	heated	subsurface	BioSep; Bandounas ligni	2013	PacBio RS, PacBio RS II	DOE JGI*	SAMN05216595	2619618868
AN70	Alphaproteobacteria	Rhizobium sp. AN70	control	surface	BioSep; Bandounas ligni	2013	Illumina NovaSeq S4	DOE JGI	SAMN33771226	2960282700
AN72	Alphaproteobacteria	Rhizoboum sp. AN72	control	surface	BioSep; Bandounas ligni	2013	ONT MinION	DeAngelis Lab	SAMN33771233	2929586531
AN73	Alphaproteobacteria	Rhizobium sp. AN73	control	surface	BioSep; Bandounas ligni	2013	ONT MinION	DeAngelis Lab	SAMN33771227	2935264491
AN83	Alphaproteobacteria	Rhizoboum sp. AN83	heated	subsurface	BioSep; Bandounas ligni	2013	ONT MinION	DeAngelis Lab	SAMN33771234	2929591855
AN88	Alphaproteobacteria	Rhizoboum sp. AN88	control	subsurface	BioSep; Bandounas ligni	2013	ONT MinION	DeAngelis Lab	SAMN33771235	2929574867
AN95	Alphaproteobacteria	Rhizoboum sp. AN95	heated	subsurface	BioSep; Bandounas ligni	2013	ONT MinION	DeAngelis Lab	SAMN33771236	2930903789

Betaproteobacteria *Paraburkholderia* spp. clade

GAS32	Betaproteobacteria	Paraburkholderia sp. GAS32	heated	mineral	VL55 ROC	2014	Illumina NovaSeq S4	DOE JGI	SAMN33771245	2981470370
GAS33	Betaproteobacteria	Paraburkholderia sp. GAS33	heated	mineral	VL55 ROC	2014	Illumina NovaSeq S4	DOE JGI	SAMN33771246	2981462429
GAS38	Betaproteobacteria	Paraburkholderia sp. GAS38	heated	mineral	VL55 ROC	2014	Illumina NovaSeq S4	DOE JGI	SAMN33771249	2981752396
GAS41	Betaproteobacteria	Paraburkholderia sp. GAS41	heated	mineral	VL55 PP	2014	Illumina NovaSeq S4	DOE JGI	SAMN33771250	2981392040
GAS42	Betaproteobacteria	Paraburkholderia sp. GAS42	heated	mineral	VL55 PP	2014	Illumina NovaSeq S4	DOE JGI	SAMN33771251	2981399414
GAS82	Betaproteobacteria	Paraburkholderia sp. GAS82	control	mineral	VL55 PP	2014	Illumina NovaSeq S4	DOE JGI	SAMN33771253	2981406600
GAS86	Betaproteobacteria	Paraburkholderia phenazinium GAS86	heated	mineral	VL55 xylan	2014	PacBio RS II	DOE JGI*	SAMN05444168	2695421038
GAS95	Betaproteobacteria	Paraburkholderia phenazinium GAS95	heated	mineral	modified CMC	2014	PacBio RS II	DOE JGI*	SAMN05444165	2695420309
GAS106B	Betaproteobacteria	Paraburkholderia fungorum GAS106B	heated	mineral	modified CMC	2014	PacBio RS II	DOE JGI*	SAMN05443245	2690315676
GAS199	Betaproteobacteria	Paraburkholderia sp. GAS199	control	mineral	VL55 PP	2014	Illumina NovaSeq S4	DOE JGI	SAMN33771242	2981432666
GAS205	Betaproteobacteria	Paraburkholderia sp. GAS205	heated	mineral	VL55 PP	2014	Illumina NovaSeq S4	DOE JGI	SAMN33771243	2981440061
GAS206C	Betaproteobacteria	Paraburkholderia sp. GAS206C	heated	mineral	VL55 PP	2014	Illumina NovaSeq S4	DOE JGI	SAMN33771244	2981759879
GAS332	Betaproteobacteria	Burkholderia sp. GAS332	control	mineral	modified CMC	2014	PacBio RS II	DOE JGI*	SAMN05444172	2695420918
GAS334	Betaproteobacteria	Paraburkholderia sp. GAS334	control	mineral	VL55 PP	2014	Illumina NovaSeq S4	DOE JGI	SAMN33771247	2981447645
GAS348	Betaproteobacteria	Paraburkholderia sp. GAS348	heated	mineral	VL55 PP	2014	Illumina NovaSeq S4	DOE JGI	SAMN33771248	2981455594
GAS448	Betaproteobacteria	Paraburkholderia sp. GAS448	control	mineral	VL55 ROC	2014	Illumina NovaSeq S4	DOE JGI	SAMN33771252	2981774849

Betaproteobacteria *Ralstonia* spp. clade

AB5-6	Betaproteobacteria	Ralstonia sp. AB5-6	control	organic	1% nutrient broth	2014	ONT MinION, Illumina NextSeq 2000	DeAngelis Lab, SeqCenter	SAMN33771260	2966226929
AB8-16	Betaproteobacteria	Ralstonia sp. AB8-16	control	organic	1% nutrient broth	2014	ONT MinION, Illumina NextSeq 2000	DeAngelis Lab, SeqCenter	SAMN33771261	2966232277
AB22-23	Betaproteobacteria	Ralstonia sp. AB22-23	control	mineral	1% nutrient broth	2014	ONT MinION, Illumina NextSeq 2000	DeAngelis Lab, SeqCenter	SAMN33771254	2966237621
AB24-4	Betaproteobacteria	Ralstonia sp. AB24-4	control	mineral	1% nutrient broth	2014	ONT MinION	DeAngelis Lab	SAMN33771255	2923570319
AB28-3	Betaproteobacteria	Ralstonia sp. AB28-3	heated	mineral	1% nutrient broth	2014	ONT MinION, Illumina NextSeq 2000	DeAngelis Lab, SeqCenter	SAMN33771256	2966242759
AB28-4	Betaproteobacteria	Ralstonia sp. AB28-4	heated	mineral	1% nutrient broth	2014	ONT MinION, Illumina NextSeq 2000	DeAngelis Lab, SeqCenter	SAMN33771257	2966248084
AB36-4	Betaproteobacteria	Ralstonia sp. AB36-4	heated	mineral	1% nutrient broth	2014	ONT MinION	DeAngelis Lab	SAMN33771259	2928862943
AB36-13C	Betaproteobacteria	Ralstonia sp. AB36-13C	heated	mineral	1% nutrient broth	2014	ONT MinION, Illumina NextSeq 2000	DeAngelis Lab, SeqCenter	SAMN33771258	2966253427
GP71	Betaproteobacteria	Ralstonia sp. GP71	control	organic	VL55 PP	2014	ONT MinION	DeAngelis Lab	SAMN33771268	2923564856
GP73	Betaproteobacteria	Ralstonia sp. GP73	control	mineral	water agar	2014	ONT MinION	DeAngelis Lab	SAMN33771269	2923725988
GP101	Betaproteobacteria	Ralstonia sp. GP101	heated	organic	VL55 PP	2014	ONT MinION	DeAngelis Lab	SAMN33771262	2923597253
GP103	Betaproteobacteria	Ralstonia sp. GP103	heated	organic	VL55 PP	2014	ONT MinION, Illumina NextSeq 2000	DeAngelis Lab, SeqCenter	SAMN33771263	2966258773
GP104	Betaproteobacteria	Ralstonia sp. GP104	heated	organic	VL55 PP	2014	ONT MinION, Illumina NextSeq 2000	DeAngelis Lab, SeqCenter	SAMN33771264	2966264122

Table S1. Isolate Metadata

GP174	Betaproteobacteria	Ralstonia sp. GP174	control	organic	VL55 PP	2014	ONT MinION	DeAngelis Lab	SAMN33771265	2923731573
GP175	Betaproteobacteria	Ralstonia sp. GP175	control	mineral	water agar	2014	ONT MinION, Illumina NextSeq 2000	DeAngelis Lab, SeqCenter	SAMN33771266	2966269469
GP176	Betaproteobacteria	Ralstonia sp. GP176	control	mineral	water agar	2014	ONT MinION, Illumina NextSeq 2000	DeAngelis Lab, SeqCenter	SAMN33771267	2966274816
GPP58	Betaproteobacteria	Ralstonia sp. GPP58	heated	mineral	VL55 PP	2014	ONT MinION	DeAngelis Lab	SAMN33771270	2927050446
GPP79	Betaproteobacteria	Ralstonia sp. GPP79	heated	mineral	VL55 PP	2014	ONT MinION	DeAngelis Lab	SAMN33771271	2923720395

Table S2. Genome Metadata

Genome	Clade	Treatment	Contigs	Total Length	% G+C	% Completion	% Redundancy	Confidence	Genes	Ave Gene Length	Genes per Kb	Singleton GCs	Total GCs	16S rRNA Genes	CAZymes
GAS1054	Kitasatospora	control	2	9526358	72.3	100	4.23	1	8632	945	0.91	643	7308	10	326
GAS204B	Kitasatospora	heated	84	8423638	71.9	98.6	8.45	1	7319	1014	0.87	47	6790	1	297
GP157	Kitasatospora	heated	4	8969559	71.5	98.6	9.86	1	8156	954	0.91	19	7408	10	281
GP160	Kitasatospora	heated	2	8159895	72.3	98.6	2.82	1	6762	1051	0.83	298	6127	10	240
GP163	Kitasatospora	heated	5	8638857	71.8	98.6	7.04	0.9	7588	990	0.88	50	6841	9	308
GP28	Kitasatospora	heated	4	8987849	71.5	97.2	9.86	1	8618	890	0.96	85	7471	10	306
GP30	Kitasatospora	heated	110	8861088	71.6	98.6	7.04	1	7986	973	0.90	19	7399	1	271
GP31	Kitasatospora	heated	2	9246900	72.7	98.6	8.45	1	7975	1014	0.86	908	7229	10	307
GP36	Kitasatospora	heated	8	8071464	71.5	97.2	12.7	1	7918	867	0.98	88	6758	10	274
GP50	Kitasatospora	control	2	8590657	71.8	98.6	8.45	1	7578	988	0.88	60	6846	9	299
GP55	Kitasatospora	heated	3	8962713	71.5	98.6	7.04	1	7990	979	0.89	3	7388	10	270
GP82	Kitasatospora	heated	138	8204036	70.7	100	8.45	1	7384	938	0.90	1599	6746	1	264
MAA18	Kitasatospora	heated	81	8759053	71.7	98.6	7.04	1	7764	988	0.89	121	7170	1	267
MAA19	Kitasatospora	heated	96	9091641	72.6	100	7.04	1	8119	968	0.89	437	7468	1	289
MAA2	Kitasatospora	heated	6	9209497	71.4	98.6	7.04	1	8532	928	0.93	360	7627	10	288
MAA36	Kitasatospora	control	3	7481034	73.1	98.6	5.63	1	7089	866	0.95	1061	6306	10	256
MAA4	Kitasatospora	heated	71	8171757	71.7	98.6	7.04	1	7330	998	0.90	998	6713	1	284
MAA52	Kitasatospora	control	3	8580784	71.8	98.6	8.45	1	7624	973	0.89	48	6804	9	304
MAA75	Kitasatospora	heated	2	8524674	71.8	100	8.45	1	7538	984	0.88	190	6886	9	297
MAA81	Kitasatospora	control	5	8784971	72.2	100	7.04	1	8106	935	0.92	1096	7063	9	322
MAP12-15	Kitasatospora	heated	100	8934013	71.5	98.6	8.45	1	7785	1018	0.87	3	7147	1	296
MAP12-4	Kitasatospora	heated	6	8976678	72.5	100	7.04	1	8514	882	0.95	457	7381	10	317
MAP12-44	Kitasatospora	heated	4	9043878	71.4	98.6	8.45	1	7830	1019	0.87	17	7149	10	298
MAP12-9	Kitasatospora	heated	72	8102036	71.6	98.6	4.23	1	7055	1019	0.87	1	6553	1	263
MAP2-59	Kitasatospora	heated	9	8170709	71.1	100	11.3	1	7476	943	0.91	281	6705	9	355
MAP5-34	Kitasatospora	heated	118	7318731	71.2	98.6	9.86	1	6448	985	0.88	982	5959	1	262
MAP5-40	Kitasatospora	heated	7	7915022	71.2	97.2	14.1	0.9	7635	880	0.96	178	6643	9	393
MAP8-42	Kitasatospora	heated	12	7813028	71.2	100	12.7	1	7149	946	0.92	92	6428	9	371
GAS138	Bradyrhizobium	control	1	9092036	61.4	100	2.82	1	8434	883	0.93	1663	7532	1	206
GAS165	Bradyrhizobium	control	3	6132387	62.5	100	2.82	1	5820	916	0.95	702	5492	1	155
GAS231	Bradyrhizobium	control	1	7584136	62.6	100	2.82	1	7021	933	0.93	11	6516	1	182
GAS242	Bradyrhizobium	heated	1	9184651	61.9	100	0	1	8622	876	0.94	1401	7637	1	202
GAS366	Bradyrhizobium	heated	1	7583959	62.6	100	8.45	1	7240	897	0.95	74	6564	1	188

Table S2. Genome Metadata

Genome	Clade	Treatment	Contigs	Total Length	% G+C	% Completion	% Redundancy	Confidence	Genes	Ave Gene Length	Genes per Kb	Singleton GCs	Total GCs	16S rRNA Genes	CAZymes
GAS369	Bradyrhizobium	heated	1	7841944	60.9	100	7.04	1	7252	919	0.92	261	6737	1	174
GAS401	Bradyrhizobium	heated	1	7525117	61.2	100	2.82	1	7107	893	0.94	1492	6508	1	184
GAS478	Bradyrhizobium	control	4	11738562	61.4	100	2.82	1	11048	850	0.94	2137	8725	1	231
GAS499	Bradyrhizobium	control	1	7909999	61.8	100	0	1	7330	917	0.93	954	6766	1	213
GAS522	Bradyrhizobium	control	2	8269569	62.3	100	8.45	1	7801	896	0.94	67	6980	1	187
GAS524	Bradyrhizobium	control	1	8339115	62.3	100	8.45	1	7921	890	0.95	136	7106	1	186
GAS525	Bradyrhizobium	control	1	7951160	60.8	100	4.23	1	7353	920	0.92	417	6847	1	174
MAP5-43	Bradyrhizobium	heated	1	8047392	62.1	98.6	1.41	1	7660	888	0.95	1300	6918	2	189
MT12	Bradyrhizobium	control	2	8967172	63.8	100	5.63	1	8411	905	0.94	1741	7703	1	212
MT34	Bradyrhizobium	control	1	8150868	62.3	100	7.04	1	7657	901	0.94	165	6901	1	190
AN5	Rhizobium	control	5	5527901	58.6	100	0	1	5255	931	0.95	412	5095	4	173
AN63	Rhizobium	heated	4	5617997	58.6	98.6	1.41	1	5767	851	1.03	30	5143	4	191
AN64	Rhizobium	control	3	5639358	58.5	100	0	1	5413	917	0.96	30	5119	5	179
AN67	Rhizobium	control	4	5619457	58.6	98.6	0	1	5885	830	1.05	54	5163	4	204
AN68	Rhizobium	control	3	5639523	58.5	100	1.41	1	5375	924	0.95	19	5106	5	176
AN69	Rhizobium	control	4	5618682	58.6	100	1.41	1	5834	839	1.04	30	5147	4	199
AN6A	Rhizobium	heated	4	5625739	58.6	100	0	1	5241	950	0.93	10	5036	4	173
AN70	Rhizobium	control	22	5208341	59.2	100	1.41	1	4904	945	0.94	13	4750	1	158
AN72	Rhizobium	control	3	5470029	58.8	100	0	1	5144	938	0.94	198	4906	4	176
AN73	Rhizobium	control	3	5252559	59.1	100	2.82	1	5030	921	0.96	37	4771	5	166
AN83	Rhizobium	heated	4	5617845	58.6	94.4	2.82	1	5748	851	1.02	42	5134	4	198
AN88	Rhizobium	control	3	5642017	58.5	100	0	1	5420	916	0.96	26	5120	5	176
AN95	Rhizobium	heated	4	5616373	58.6	97.2	1.41	1	5888	829	1.05	50	5160	4	206
28DA2	Rhizobium	heated	4	5618214	58.6	95.8	1.41	1	5793	845	1.03	46	5159	4	192
GAS106B	Paraburkholderia	heated	4	8581175	61.2	98.6	1.41	1	7431	974	0.87	1248	6882	8	230
GAS199	Paraburkholderia	control	20	8196845	62.4	100	1.41	1	7166	986	0.87	992	6569	1	216
GAS205	Paraburkholderia	heated	35	8284543	61.4	100	4.23	1	7360	975	0.89	1	6848	0	221
GAS206C	Paraburkholderia	heated	30	8284727	61.4	100	4.23	1	7353	976	0.89	1	6844	0	221
GAS32	Paraburkholderia	heated	141	10460609	61.1	100	1.41	1	9732	905	0.93	1450	8485	1	239
GAS33	Paraburkholderia	heated	38	8627607	61.3	98.6	4.23	1	7694	968	0.89	407	7118	0	228
GAS332	Paraburkholderia	control	3	10416547	61.4	100	2.82	1	9124	972	0.88	1511	8113	7	257
GAS334	Paraburkholderia	control	102	8203652	61.6	100	1.41	1	7624	904	0.93	719	6714	1	185
GAS348	Paraburkholderia	heated	79	7262714	61.2	100	2.82	1	6476	942	0.89	829	5856	1	182

Table S2. Genome Metadata

Genome	Clade	Treatment	Contigs	Total Length	% G+C	% Completion	% Redundancy	Confidence	Genes	Ave Gene Length	Genes per Kb	Singleton GCs	Total GCs	16S rRNA Genes	CAZymes
GAS38	Paraburkholderia	heated	34	8268867	63.0	100	4.23	1	7262	987	0.88	255	6788	1	223
GAS41	Paraburkholderia	heated	92	7839433	61.8	100	2.82	1	7094	937	0.90	838	6400	1	198
GAS42	Paraburkholderia	heated	69	7534538	61.7	100	1.41	1	6921	923	0.92	557	6261	1	176
GAS448	Paraburkholderia	control	168	10103218	61.9	100	1.41	1	9458	894	0.94	1816	8202	1	243
GAS82	Paraburkholderia	control	31	8375761	61.4	100	4.23	1	7415	979	0.89	309	6885	0	227
GAS86	Paraburkholderia	heated	2	8319467	61.4	100	4.23	1	7256	988	0.87	161	6729	5	224
GAS95	Paraburkholderia	heated	3	8526942	62.8	100	4.23	1	7407	992	0.87	249	6855	6	228
AB22-23	Ralstonia	control	7	5298867	63.6	94.4	2.82	0.9	4960	945	0.94	2	4724	3	117
AB24-4	Ralstonia	control	4	5432636	63.6	98.6	2.82	1	5236	906	0.96	21	4861	3	125
AB28-3	Ralstonia	heated	6	5483600	63.6	100	2.82	1	5133	944	0.94	1	4911	3	121
AB28-4	Ralstonia	heated	5	5513759	63.6	100	2.82	1	5159	945	0.94	0	4913	3	121
AB36-13C	Ralstonia	heated	5	5513759	63.6	100	2.82	1	5159	945	0.94	0	4912	3	121
AB36-4	Ralstonia	heated	6	5579439	63.5	100	5.63	1	5304	923	0.95	11	4916	3	122
AB5-6	Ralstonia	control	5	5513753	63.6	100	2.82	1	5164	944	0.94	0	4914	3	121
AB8-16	Ralstonia	control	5	5513543	63.6	100	2.82	1	5159	945	0.94	0	4913	3	121
GP101	Ralstonia	heated	5	5513656	63.6	98.6	4.23	1	5359	899	0.97	21	4912	3	125
GP103	Ralstonia	heated	6	5515304	63.6	100	2.82	1	5162	944	0.94	0	4914	3	121
GP104	Ralstonia	heated	6	5515238	63.6	100	2.82	1	5161	944	0.94	0	4914	3	121
GP174	Ralstonia	control	5	5513800	63.6	98.6	4.23	1	5352	899	0.97	25	4926	3	126
GP175	Ralstonia	control	6	5515256	63.6	100	2.82	1	5162	944	0.94	0	4914	3	121
GP176	Ralstonia	control	18	5553901	63.6	100	2.82	1	5211	942	0.94	24	4940	3	121
GP71	Ralstonia	control	4	5432770	63.6	98.6	4.23	1	5201	915	0.96	15	4851	3	126
GP73	Ralstonia	control	5	5477586	63.6	98.6	5.63	1	5311	899	0.97	16	4908	3	129
GPP58	Ralstonia	heated	5	5513689	63.6	98.6	8.45	1	5346	902	0.97	23	4931	3	125
GPP79	Ralstonia	heated	5	5513696	63.6	98.6	4.23	1	5318	906	0.96	17	4921	3	126

Table S3. MinION Assembly Stats

Isolate	Phylum	Clade	Assembly Method	Coverage (X)	CheckM Lineage	Assembly Length	% G+C	Contigs	N50	Completeness	Contamination
GAS1054	Actinobacteria	Kitasatospora	de novo	37.8	o__Actinomycetales	9526358	72.3	2	9465601	95.7	2.9
GP28	Actinobacteria	Kitasatospora	de novo	40.1	o__Actinomycetales	8987849	71.5	4	8885772	98.2	0.8
GP31	Actinobacteria	Kitasatospora	de novo	38.9	o__Actinomycetales	9246900	72.7	2	9218386	99.3	3.2
GP36	Actinobacteria	Kitasatospora	de novo	44.6	o__Actinomycetales	8071464	71.5	8	2279891	95.0	0.8
GP50	Actinobacteria	Kitasatospora	de novo	41.9	o__Actinomycetales	8590657	71.8	2	8097869	99.5	0.9
GP157	Actinobacteria	Kitasatospora	de novo	40.1	o__Actinomycetales	8969559	71.5	4	8878309	99.5	0.8
GP160	Actinobacteria	Kitasatospora	de novo	44.1	o__Actinomycetales	8159895	72.3	2	6036722	99.0	3.4
GP163	Actinobacteria	Kitasatospora	de novo	41.7	o__Actinomycetales	8638857	71.8	5	8084037	99.0	0.9
MAA2	Actinobacteria	Kitasatospora	de novo	39.1	o__Actinomycetales	9209497	71.4	6	8574763	97.6	1.6
MAA36	Actinobacteria	Kitasatospora	de novo	42.8	o__Actinomycetales	7481034	73.1	3	7229582	94.7	2.1
MAA52	Actinobacteria	Kitasatospora	de novo	47.2	o__Actinomycetales	8580784	71.8	3	7998586	98.4	1.4
MAA75	Actinobacteria	Kitasatospora	de novo	42.2	o__Actinomycetales	8524674	71.8	2	8012833	100.0	1.4
MAA81	Actinobacteria	Kitasatospora	de novo	41.0	o__Actinomycetales	8784971	72.2	5	8512193	97.0	0.7
MAP2-59	Actinobacteria	Kitasatospora	de novo	44.1	o__Actinomycetales	8170709	71.1	9	8011956	99.5	1.2
MAP5-40	Actinobacteria	Kitasatospora	de novo	45.5	o__Actinomycetales	7915022	71.2	7	7591491	97.6	0.6
MAP8-42	Actinobacteria	Kitasatospora	de novo	46.1	o__Actinomycetales	7813028	71.2	12	6256654	98.8	0.6
MAP12-4	Actinobacteria	Kitasatospora	de novo	45.1	o__Actinomycetales	8976678	72.5	6	8304589	96.9	2.4
GAS366	Alphaproteobacteria	Bradyrhizobium	de novo	32.0	f__Bradyrhizobiaceae	7583959	62.6	1	7583959	99.3	1.4
MAP5-43	Alphaproteobacteria	Bradyrhizobium	de novo	41.9	f__Bradyrhizobiaceae	8047392	62.1	1	8047392	99.3	0.9
28DA2	Alphaproteobacteria	Rhizobium	de novo	32.0	f__Rhizobiaceae	5618214	58.6	4	2869860	96.8	0.1
AN63	Alphaproteobacteria	Rhizobium	de novo	44.9	f__Rhizobiaceae	5617997	58.6	4	2869899	97.4	0.1
AN64	Alphaproteobacteria	Rhizobium	de novo	31.9	f__Rhizobiaceae	5639358	58.5	3	2110257	99.2	0.8
AN67	Alphaproteobacteria	Rhizobium	de novo	44.8	f__Rhizobiaceae	5619457	58.6	4	2870042	97.6	0.1
AN68	Alphaproteobacteria	Rhizobium	de novo	44.7	f__Rhizobiaceae	5639523	58.5	3	2110415	99.6	0.7
AN69	Alphaproteobacteria	Rhizobium	de novo	44.9	f__Rhizobiaceae	5618682	58.6	4	2870003	97.8	0.1
AN72	Alphaproteobacteria	Rhizobium	de novo	32.9	f__Rhizobiaceae	5470029	58.8	3	2915437	99.8	0.6
AN73	Alphaproteobacteria	Rhizobium	de novo	48.0	f__Rhizobiaceae	5252559	59.2	3	2912879	99.5	0.7
AN83	Alphaproteobacteria	Rhizobium	de novo	32.0	f__Rhizobiaceae	5617845	58.6	4	2869877	98.6	0.1
AN88	Alphaproteobacteria	Rhizobium	de novo	31.9	f__Rhizobiaceae	5642017	58.5	3	2112953	98.7	0.8
AN95	Alphaproteobacteria	Rhizobium	de novo	44.9	f__Rhizobiaceae	5616373	58.6	4	2870026	97.3	0.1
AB5-6	Betaproteobacteria	Ralstonia	hybrid	174.7	f__Burkholderiaceae	5513753	63.6	5	3537649	99.9	0.5
AB8-16	Betaproteobacteria	Ralstonia	hybrid	146.8	f__Burkholderiaceae	5513543	63.6	5	3537481	99.9	0.5
AB22-23	Betaproteobacteria	Ralstonia	hybrid	184.9	f__Burkholderiaceae	5298867	63.6	7	1386635	92.6	0.5
AB24-4	Betaproteobacteria	Ralstonia	de novo	39.8	f__Burkholderiaceae	5432636	63.6	4	3537574	99.0	0.5

Table S3. MinION Assembly Stats

Isolate	Phylum	Clade	Assembly Method	Coverage (X)	CheckM Lineage	Assembly Length	% G+C	Contigs	N50	Completeness	Contamination
AB36-4	Betaproteobacteria	Ralstonia	de novo	38.7	f__Burkholderiaceae	5579439	63.5	6	3564194	98.8	0.5
GP71	Betaproteobacteria	Ralstonia	de novo	39.8	f__Burkholderiaceae	5432770	63.6	4	3537633	99.1	0.5
GP73	Betaproteobacteria	Ralstonia	de novo	39.4	f__Burkholderiaceae	5477586	63.6	5	3537750	97.9	0.5
GP101	Betaproteobacteria	Ralstonia	de novo	39.2	f__Burkholderiaceae	5513656	63.6	5	3537632	98.9	0.5
GP104	Betaproteobacteria	Ralstonia	hybrid	187.3	f__Burkholderiaceae	5515238	63.6	6	3537649	99.9	0.5
GP174	Betaproteobacteria	Ralstonia	de novo	39.2	f__Burkholderiaceae	5513800	63.6	5	3537701	98.2	0.5
GP176	Betaproteobacteria	Ralstonia	hybrid	190.9	f__Burkholderiaceae	5553306	63.6	16	1386635	99.9	0.5
GPP58	Betaproteobacteria	Ralstonia	de novo	39.2	f__Burkholderiaceae	5513689	63.6	5	3537615	98.5	0.5
GPP79	Betaproteobacteria	Ralstonia	de novo	39.2	f__Burkholderiaceae	5512301	63.6	5	3537617	98.7	0.5

Table S4. KOfam Functional Enrichment

Description	Enrichment Score	P-value	Q-value	Associated Treatment	KO Identifier	Gene Cluster IDs	p_Heated	p_Control	Clade
nicotinamide mononucleotide transporter	9.91	0.002	0.75	heated	K03811	GC_00002777	1.0	0.6	Kitasatospora
gentisate 1,2-dioxygenase [EC:1.13.11.4]	8.12	0.004	0.75	control	K00450	GC_00005029	0.3	1.0	Kitasatospora
(5-formylfuran-3-yl)methyl phosphate synthase [EC:4.2.3.153]	8.12	0.004	0.75	control	K09733	GC_00002980	0.3	1.0	Kitasatospora
pentalenene oxygenase [EC:1.14.15.32]	8.12	0.004	0.75	control	K15907	GC_00006887, GC_00010110, GC_00016724, GC_00029715	0.3	1.0	Kitasatospora
Rrf2 family transcriptional regulator, repressor of oqxAB	7.89	0.005	0.75	control	K19587	GC_00007190	0.2	0.8	Kitasatospora
cell division protease FtsH [EC:3.4.24.-]	7.02	0.008	0.75	control	K03798	GC_00005770, GC_00006673	0.3	1.0	Kitasatospora
thiamine kinase [EC:2.7.1.89]	7.02	0.008	0.75	control	K07251	GC_00004527	0.3	1.0	Kitasatospora
toxin FitB [EC:3.1.-.-]	7.02	0.008	0.75	control	K07062	GC_00004283, GC_00020036	0.3	1.0	Kitasatospora
maleate isomerase [EC:5.2.1.1]	7.02	0.008	0.75	control	K01799	GC_00005486, GC_00028536, GC_00028579	0.3	1.0	Kitasatospora
polysaccharide biosynthesis protein PslG	6.39	0.011	0.75	control	K21000	GC_00007371, GC_00017393	0.2	0.8	Kitasatospora
magnesium-dependent phosphatase 1 [EC:3.1.3.48 3.1.3.-]	6.39	0.011	0.75	control	K17619	GC_00008076, GC_00017122	0.2	0.8	Kitasatospora
arsenite oxidase small subunit [EC:1.20.2.1 1.20.9.1]	6.39	0.011	0.75	control	K08355	GC_00006620	0.2	0.8	Kitasatospora
acyl-CoA oxidase [EC:1.3.3.6]	6.39	0.011	0.75	control	K00232	GC_00007179, GC_00009968, GC_00013461, GC_00023709	0.2	0.8	Kitasatospora
RpiR family transcriptional regulator, carbohydrate utilization regulator	6.39	0.011	0.75	control	K19337	GC_00006408	0.2	0.8	Kitasatospora
small membrane protein	6.39	0.011	0.75	control	K09153	GC_00006341	0.2	0.8	Kitasatospora
antitoxin Phd	6.39	0.011	0.75	heated	K19165	GC_00003864, GC_00009763	0.8	0.2	Kitasatospora
dimethylglycine oxidase [EC:1.5.3.10]	6.39	0.011	0.75	heated	K00309	GC_00003488	0.8	0.2	Kitasatospora
glutamate transport system ATP-binding protein [EC:7.4.2.1]	6.39	0.011	0.75	heated	K10008	GC_00000458	0.8	0.2	Kitasatospora
glutamate transport system substrate-binding protein	6.39	0.011	0.75	heated	K10005	GC_00002724	0.8	0.2	Kitasatospora
serine O-acetyltransferase [EC:2.3.1.30]	6.09	0.014	0.75	heated	K00640	GC_00004630, GC_00025323, GC_00029649	0.6	0.0	Kitasatospora
NTF2-related export protein 1/2	5.46	0.019	0.75	control	K14285	GC_00015502	0.0	0.4	Kitasatospora
heme exporter protein B	5.46	0.019	0.75	control	K02194	GC_00016395	0.0	0.4	Kitasatospora
L-proline 4-hydroxylase [EC:1.14.11.57]	5.46	0.019	0.75	control	K21615	GC_00024079, GC_00025558, GC_00028750	0.0	0.4	Kitasatospora
prephenate decarboxylase [EC:4.1.1.100]	5.46	0.019	0.75	control	K19546	GC_00016699	0.0	0.4	Kitasatospora
damage-control phosphatase, subfamily III [EC:3.1.3.-]	5.46	0.019	0.75	heated	K23114	GC_00003704, GC_00008583	1.0	0.6	Kitasatospora
zinc/manganese transport system ATP-binding protein	5.46	0.019	0.75	heated	K02074	GC_00000338	1.0	0.6	Kitasatospora
pilus assembly protein CpaB	5.46	0.019	0.75	heated	K02279	GC_00003359, GC_00009250, GC_00017467	1.0	0.6	Kitasatospora
phosphoenolpyruvate phosphomutase [EC:5.4.2.9]	5.38	0.020	0.75	control	K01841	GC_00010589, GC_00028165	0.1	0.6	Kitasatospora
2-hydroxy-6-oxonona-2,4-dienedioate hydrolase [EC:3.7.1.14]	5.38	0.020	0.75	control	K05714	GC_00009176	0.1	0.6	Kitasatospora
2,3-dihydroxyphenylpropionate 1,2-dioxygenase [EC:1.13.11.16]	5.38	0.020	0.75	control	K05713	GC_00008123	0.1	0.6	Kitasatospora
phosphonopyruvate decarboxylase [EC:4.1.1.82]	5.38	0.020	0.75	control	K09459	GC_00010304, GC_00021033	0.1	0.6	Kitasatospora
hercynylcysteine S-oxide lyase [EC:4.4.1.36]	5.38	0.020	0.75	control	K18913	GC_00009766	0.1	0.6	Kitasatospora
starvation-inducible DNA-binding protein	5.38	0.020	0.75	heated	K04047	GC_00003474, GC_00025296	0.9	0.4	Kitasatospora
ATP-dependent RNA helicase DeaD [EC:3.6.4.13]	5.38	0.020	0.75	heated	K05592	GC_00003429	0.9	0.4	Kitasatospora
putative chitinase	5.28	0.022	0.75	control	K03791	GC_00003830	0.4	1.0	Kitasatospora
TetR/AcrR family transcriptional regulator, fatty acid metabolism regulator protein	5.20	0.023	0.75	control	K13770	GC_00005998	0.3	0.8	Kitasatospora

Table S4. KOfam Functional Enrichment

Description	Enrichment Score	P-value	Q-value	Associated Treatment	KO Identifier	Gene Cluster IDs	p_Heated	p_Control	Clade
alpha-N-acetylglucosaminidase [EC:3.2.1.50]	5.20	0.023	0.75	control	K01205	GC_00005322	0.3	0.8	Kitasatospora
peptidoglycan DL-endopeptidase RipA [EC:3.4.-.-]	5.20	0.023	0.75	control	K21473	GC_00005972	0.3	0.8	Kitasatospora
polyprenyl-phospho-N-acetylgalactosaminyl synthase	5.20	0.023	0.75	control	K22907	GC_00005503	0.3	0.8	Kitasatospora
protein HIRA/HIR1	5.20	0.023	0.75	control	K11293	GC_00005634, GC_00017673	0.3	0.8	Kitasatospora
colanic acid/amylovoran biosynthesis protein WcaK/AmsJ	5.20	0.023	0.75	control	K16710	GC_00007252, GC_00010530	0.3	0.8	Kitasatospora
5-carboxymethyl-2-hydroxymuconate isomerase [EC:5.3.3.10]	5.20	0.023	0.75	heated	K01826	GC_00004867, GC_00015586, GC_00016351, GC_00018400, GC_00019132	0.7	0.2	Kitasatospora
protein PhnA	5.20	0.023	0.75	heated	K06193	GC_00003716	0.7	0.2	Kitasatospora
putative ATP-dependent endonuclease of the OLD family	5.20	0.023	0.75	heated	K07459	GC_00010889, GC_00015153, GC_00016745, GC_00018242, GC_00022573, GC_00023176, GC_00024225, GC_00024358, GC_00024735, GC_00028275	0.7	0.2	Kitasatospora
polyether ionophore transport system permease protein	5.20	0.023	0.75	heated	K25149	GC_00005968, GC_00009675, GC_00017311	0.7	0.2	Kitasatospora
polyether ionophore transport system ATP-binding protein	5.20	0.023	0.75	heated	K25150	GC_00003620	0.7	0.2	Kitasatospora
gamma-polyglutamate biosynthesis protein CapC	4.77	0.029	0.75	control	K22116	GC_00030495	0.0	0.2	Kitasatospora
lactate permease	4.77	0.029	0.75	control	K03303	GC_00029165	0.0	0.2	Kitasatospora
streptogrisin C [EC:3.4.21.-]	4.77	0.029	0.75	control	K18546	GC_00021229	0.0	0.2	Kitasatospora
C3 family ADP-ribosyltransferase [EC:2.4.2.-]	4.77	0.029	0.75	control	K11044	GC_00028645	0.0	0.2	Kitasatospora
2-methylcitrate dehydratase [EC:4.2.1.79]	4.77	0.029	0.75	control	K01720	GC_00027771	0.0	0.2	Kitasatospora
ATP-dependent helicase IRC3 [EC:5.6.2.-]	4.77	0.029	0.75	control	K17677	GC_00027236	0.0	0.2	Kitasatospora
iron(III)-enterobactin esterase [EC:3.1.1.108]	4.77	0.029	0.75	control	K07214	GC_00030704	0.0	0.2	Kitasatospora
gamma-polyglutamate synthase [EC:6.3.2.-]	4.77	0.029	0.75	control	K01932	GC_00021332	0.0	0.2	Kitasatospora
cobalamin transport system substrate-binding protein	4.77	0.029	0.75	control	K25034	GC_00025894	0.0	0.2	Kitasatospora
extracellular factor (EF) 3-hydroxypalmitic acid methyl ester biosynthesis protein	4.77	0.029	0.75	control	K19620	GC_00027366	0.0	0.2	Kitasatospora
adenosylcobinamide hydrolase [EC:3.5.1.90]	4.77	0.029	0.75	control	K08260	GC_00028111	0.0	0.2	Kitasatospora
phenylpyruvate C(3)-methyltransferase [EC:2.1.1.281]	4.77	0.029	0.75	control	K21457	GC_00026745	0.0	0.2	Kitasatospora
catechol O-methyltransferase [EC:2.1.1.6]	4.77	0.029	0.75	control	K00545	GC_00030359	0.0	0.2	Kitasatospora
(+)-beta-caryophyllene/(+)-caryolan-1-ol synthase [EC:4.2.3.89 4.2.1.138]	4.77	0.029	0.75	control	K18111	GC_00025119	0.0	0.2	Kitasatospora
ureidoacrylate peracid hydrolase [EC:3.5.1.110]	4.77	0.029	0.75	control	K09020	GC_00028512	0.0	0.2	Kitasatospora
cytochrome P450 family 20 subfamily A [EC:1.14.-.-]	4.77	0.029	0.75	control	K07435	GC_00029795	0.0	0.2	Kitasatospora
Xaa-Arg dipeptidase [EC:3.4.13.4]	4.77	0.029	0.75	control	K26141	GC_00030282	0.0	0.2	Kitasatospora
flagellin	4.77	0.029	0.75	control	K02406	GC_00028736	0.0	0.2	Kitasatospora
taurine dioxygenase [EC:1.14.11.17]	4.77	0.029	0.75	control	K03119	GC_00027188	0.0	0.2	Kitasatospora
GTP pyrophosphokinase [EC:2.7.6.5]	4.77	0.029	0.75	control	K07816	GC_00025630	0.0	0.2	Kitasatospora
Delta3-Delta2-enoyl-CoA isomerase [EC:5.3.3.8]	4.77	0.029	0.75	control	K07517	GC_00027945	0.0	0.2	Kitasatospora
AraC family transcriptional regulator, positive regulator of tynA and feaB	4.77	0.029	0.75	heated	K14063	GC_00002222, GC_00003471, GC_00004334, GC_00005247, GC_00006834, GC_00011801, GC_00014302, GC_00016293, GC_00016448, GC_00016676, GC_00017172, GC_00027737, GC_00028606, GC_00030372	1.0	0.8	Kitasatospora

Table S4. KOfam Functional Enrichment

Description	Enrichment Score	P-value	Q-value	Associated Treatment	KO Identifier	Gene Cluster IDs	p_Heated	p_Control	Clade
isobutyryl-CoA mutase small subunit [EC:5.4.99.13]	4.77	0.029	0.75	heated	K25821	GC_00002532	1.0	0.8	Kitasatospora
viologen exporter family transport system ATP-binding protein	4.77	0.029	0.75	heated	K25156	GC_00002381, GC_00028986	1.0	0.8	Kitasatospora
D-methionine transport system permease protein	4.77	0.029	0.75	heated	K02072	GC_00002546	1.0	0.8	Kitasatospora
viologen exporter family transport system permease protein	4.77	0.029	0.75	heated	K25155	GC_00002582, GC_00002599, GC_00024439, GC_00025307	1.0	0.8	Kitasatospora
ADP-dependent NAD(P)H-hydrate dehydratase / NAD(P)H-hydrate epimerase [EC:4.2.1.136 5.1.99.6]	4.77	0.029	0.75	heated	K23997	GC_00002700	1.0	0.8	Kitasatospora
16S rRNA (adenine1518-N6/adenine1519-N6)-dimethyltransferase [EC:2.1.1.182]	4.77	0.029	0.75	heated	K02528	GC_00002593	1.0	0.8	Kitasatospora
glutathione transport system ATP-binding protein	4.77	0.029	0.75	heated	K13892	GC_00000525	1.0	0.8	Kitasatospora
preprotein translocase subunit SecE	4.77	0.029	0.75	heated	K03073	GC_00002541, GC_00006353	1.0	0.8	Kitasatospora
molybdopterin adenylyltransferase [EC:2.7.7.75]	4.77	0.029	0.75	heated	K03831	GC_00002648	1.0	0.8	Kitasatospora
L-lactate dehydrogenase complex protein LldE	4.77	0.029	0.75	heated	K18928	GC_00000271	1.0	0.8	Kitasatospora
L-lactate dehydrogenase complex protein LldG	4.77	0.029	0.75	heated	K00782	GC_00003526, GC_00005866, GC_00015007	1.0	0.8	Kitasatospora
aerobic C4-dicarboxylate transport protein	4.77	0.029	0.75	heated	K11103	GC_00002438	1.0	0.8	Kitasatospora
arsenate reductase (glutaredoxin) [EC:1.20.4.1]	4.77	0.029	0.75	heated	K00537	GC_00002692	1.0	0.8	Kitasatospora
endoglycosylceramidase [EC:3.2.1.123]	4.77	0.029	0.75	heated	K05991	GC_00003040, GC_00016243	1.0	0.8	Kitasatospora
copper transport protein	4.77	0.029	0.75	heated	K14166	GC_00000505, GC_00011661	1.0	0.8	Kitasatospora
ribulose-phosphate 3-epimerase [EC:5.1.3.1]	4.77	0.029	0.75	heated	K01783	GC_00002559, GC_00030699	1.0	0.8	Kitasatospora
cell division protein FtsL	4.77	0.029	0.75	heated	K03586	GC_00002712, GC_00003115, GC_00018835	1.0	0.8	Kitasatospora
ATP synthase protein I	4.77	0.029	0.75	heated	K02116	GC_00002555, GC_00019837	1.0	0.8	Kitasatospora
O-acetylserine/cysteine efflux transporter	4.77	0.029	0.75	heated	K15268	GC_00002719, GC_00003350, GC_00003980, GC_00027552	1.0	0.8	Kitasatospora
branched-chain amino acid transport system substrate-binding protein	4.77	0.029	0.75	heated	K01999	GC_00002608, GC_00011727, GC_00012062, GC_00012931, GC_00022008	1.0	0.8	Kitasatospora
TetR/AcrR family transcriptional regulator, regulator of biofilm formation and stress response	4.77	0.029	0.75	heated	K23778	GC_00004758, GC_00004805, GC_00005278, GC_00005352, GC_00005852, GC_00006167, GC_00014423, GC_00017419	1.0	0.8	Kitasatospora
thiamine-phosphate pyrophosphorylase [EC:2.5.1.3]	4.77	0.029	0.75	heated	K00788	GC_00002677	1.0	0.8	Kitasatospora
endoribonuclease LACTB2 [EC:3.1.27.-]	4.77	0.029	0.75	heated	K16639	GC_00001308	1.0	0.8	Kitasatospora
NAD+ diphosphatase [EC:3.6.1.22]	4.77	0.029	0.75	heated	K03426	GC_00002612, GC_00016870	1.0	0.8	Kitasatospora
heme oxygenase (mycobilin-producing) [EC:1.14.99.57]	4.77	0.029	0.75	heated	K21481	GC_00000560	1.0	0.8	Kitasatospora
starch synthase (maltosyl-transferring) [EC:2.4.99.16]	4.77	0.029	0.75	heated	K16147	GC_00000219	1.0	0.8	Kitasatospora
O-acetyl-ADP-ribose deacetylase [EC:3.1.1.106]	4.77	0.029	0.75	heated	K23518	GC_00002654	1.0	0.8	Kitasatospora
nitrite reductase (NADH) large subunit [EC:1.7.1.15]	4.57	0.033	0.78	heated	K00362	GC_00004972	0.5	0.0	Kitasatospora
lanthionine-containing peptide SapB	4.57	0.033	0.78	heated	K24913	GC_00005053	0.5	0.0	Kitasatospora
uroporphyrinogen-III synthase [EC:4.2.1.75]	4.57	0.033	0.78	heated	K01719	GC_00005173, GC_00012627	0.5	0.0	Kitasatospora
calicheamicin 3'-O-methyl-rhamnosyltransferase [EC:2.4.1.-]	4.57	0.033	0.78	heated	K21263	GC_00005620, GC_00009704	0.5	0.0	Kitasatospora
NDP-mannose synthase	4.57	0.033	0.78	control	K21210	GC_00004251	0.5	1.0	Kitasatospora
NDP-hexose 4,6-dehydratase	4.57	0.033	0.78	control	K21211	GC_00004270	0.5	1.0	Kitasatospora
ketoreductase [EC:1.1.1.-]	4.57	0.033	0.78	control	K12420	GC_00005945, GC_00009532, GC_00021125	0.5	1.0	Kitasatospora

Table S4. KOfam Functional Enrichment

Description	Enrichment Score	P-value	Q-value	Associated Treatment	KO Identifier	Gene Cluster IDs	p_Heated	p_Control	Clade
NitT/TauT family transport system permease protein	4.57	0.033	0.78	control	K02050	GC_00004322, GC_00011643	0.5	1.0	Kitasatospora
acetyl-CoA carboxylase, biotin carboxylase subunit [EC:6.4.1.2 6.3.4.14]	4.23	0.040	0.92	control	K01961	GC_00005507	0.3	0.8	Kitasatospora
spermidine/putrescine transport system permease protein	4.23	0.040	0.92	control	K11071	GC_00005431, GC_00005532	0.3	0.8	Kitasatospora
spermidine/putrescine transport system ATP-binding protein [EC:7.6.2.11]	4.23	0.040	0.92	control	K11072	GC_00005558	0.3	0.8	Kitasatospora
4,5-DOPA dioxygenase extradiol [EC:1.13.11.-]	4.23	0.040	0.92	control	K15777	GC_00010082, GC_00011523	0.3	0.8	Kitasatospora
D-amino-acid oxidase [EC:1.4.3.3]	3.98	0.046	0.95	control	K00273	GC_00008207	0.2	0.6	Kitasatospora
lantibiotic bacteriocin	3.98	0.046	0.95	control	K20482	GC_00006294	0.2	0.6	Kitasatospora
adenylate cyclase [EC:4.6.1.1]	3.98	0.046	0.95	control	K01768	GC_00011602, GC_00016893, GC_00023100	0.2	0.6	Kitasatospora
esterase FrsA [EC:3.1.-.-]	3.98	0.046	0.95	control	K11750	GC_00010997, GC_00020805	0.2	0.6	Kitasatospora
aminocarboxymuconate-semialdehyde decarboxylase [EC:4.1.1.45]	3.98	0.046	0.95	heated	K03392	GC_00004626, GC_00008818	0.8	0.4	Kitasatospora
choline dehydrogenase [EC:1.1.99.1]	3.98	0.046	0.95	heated	K00108	GC_00003646, GC_00029120	0.8	0.4	Kitasatospora
2-phosphosulfolactate phosphatase [EC:3.1.3.71]	3.94	0.047	0.95	heated	K05979	GC_00005869, GC_00008057	0.5	0.0	Kitasatospora
assimilatory nitrate reductase catalytic subunit [EC:1.7.99.-]	3.94	0.047	0.95	heated	K00372	GC_00005201	0.5	0.0	Kitasatospora
assimilatory nitrate reductase electron transfer subunit [EC:1.7.99.	3.94	0.047	0.95	heated	K00360	GC_00005371	0.5	0.0	Kitasatospora
nitrite reductase (NADH) small subunit [EC:1.7.1.15]	3.94	0.047	0.95	heated	K00363	GC_00005380	0.5	0.0	Kitasatospora
CRISPR system Cascade subunit CasD	3.94	0.047	0.95	control	K19125	GC_00006893, GC_00007159, GC_00008758, GC_00017209	0.5	1.0	Kitasatospora
superoxide dismutase, Fe-Mn family [EC:1.15.1.1]	3.94	0.047	0.95	control	K04564	GC_00004131	0.5	1.0	Kitasatospora
erythromycin esterase [EC:3.1.1.-]	3.94	0.047	0.95	control	K06880	GC_00004004, GC_00012095, GC_00016333, GC_00028112, GC_00029602	0.5	1.0	Kitasatospora
iron uptake system component EfeO	3.94	0.047	0.95	control	K07224	GC_00004752, GC_00016137	0.5	1.0	Kitasatospora
adenylate cyclase, class 2 [EC:4.6.1.1]	3.94	0.047	0.95	control	K05873	GC_00003152, GC_00030091	0.5	1.0	Kitasatospora
N-acyl-D-amino-acid deacylase [EC:3.5.1.81]	3.94	0.047	0.95	control	K06015	GC_00003995	0.5	1.0	Kitasatospora
mercuric ion transport protein	7.50	0.006	1.00	control	K19058	GC_00004249, GC_00004888	0.4	1.0	Bradyrhizobium
MerR family transcriptional regulator, mercuric resistance operon regulatory protein	6.56	0.010	1.00	control	K08365	GC_00005825, GC_00017188, GC_00020937	0.0	0.7	Bradyrhizobium
aldose sugar dehydrogenase [EC:1.1.5.-]	5.00	0.025	1.00	control	K21430	GC_00004609	0.2	0.8	Bradyrhizobium
flavin prenyltransferase [EC:2.5.1.129]	5.00	0.025	1.00	heated	K03186	GC_00006040, GC_00009484, GC_00010750	0.8	0.2	Bradyrhizobium
N-acetylglucosaminyl-diphospho-decaprenol L-rhamnosyltransferase [EC:2.4.1.289]	5.00	0.025	1.00	control	K16870	GC_00005966, GC_00019054, GC_00024041, GC_00024221	0.0	0.6	Bradyrhizobium
oleate hydratase [EC:4.2.1.53]	4.62	0.032	1.00	heated	K10254	GC_00014562, GC_00024883	0.4	0.0	Bradyrhizobium
glucosyl-3-phosphoglycerate synthase [EC:2.4.1.266]	4.62	0.032	1.00	heated	K13693	GC_00012003	0.4	0.0	Bradyrhizobium
decaprenyl-phosphate phosphoribosyltransferase [EC:2.4.2.45]	4.62	0.032	1.00	heated	K14136	GC_00016509, GC_00018289	0.4	0.0	Bradyrhizobium
carboxylesterase [EC:3.1.1.1]	4.62	0.032	1.00	heated	K03928	GC_00011598	0.4	0.0	Bradyrhizobium
alcohol dehydrogenase [EC:1.1.1.-]	4.62	0.032	1.00	heated	K18369	GC_00020023, GC_00022054	0.4	0.0	Bradyrhizobium
ribonucleoside-diphosphate reductase beta chain [EC:1.17.4.1]	4.62	0.032	1.00	heated	K00526	GC_00013345	0.4	0.0	Bradyrhizobium
4-aminobutyrate---pyruvate transaminase [EC:2.6.1.96]	4.62	0.032	1.00	heated	K16871	GC_00012424	0.4	0.0	Bradyrhizobium
(R,R)-butanediol dehydrogenase / meso-butanediol dehydrogenase / diacetyl reductase [EC:1.1.1.4 1.1.1.- 1.1.1.303]	4.62	0.032	1.00	heated	K00004	GC_00019124, GC_00022146	0.4	0.0	Bradyrhizobium

Table S4. KOfam Functional Enrichment

Description	Enrichment Score	P-value	Q-value	Associated Treatment	KO Identifier	Gene Cluster IDs	p_Heated	p_Control	Clade
peptidoglycan LD-endopeptidase CwlK [EC:3.4.-.-]	4.62	0.032	1.00	heated	K17733	GC_00012620, GC_00020628	0.4	0.0	Bradyrhizobium
vitamin B12 transporter	4.62	0.032	1.00	heated	K16092	GC_00011777	0.4	0.0	Bradyrhizobium
pyrimidine oxygenase [EC:1.14.99.46]	4.62	0.032	1.00	heated	K09018	GC_00018396, GC_00023840	0.4	0.0	Bradyrhizobium
succinyl-CoA:acetate CoA-transferase [EC:2.8.3.18]	4.62	0.032	1.00	heated	K18118	GC_00012404	0.4	0.0	Bradyrhizobium
MFS transporter, Spinster family, sphingosine-1-phosphate transporter	4.62	0.032	1.00	control	K23677	GC_00003539, GC_00009082, GC_00009895	0.6	1.0	Bradyrhizobium
phytanoyl-CoA hydroxylase [EC:1.14.11.18]	4.62	0.032	1.00	control	K00477	GC_00003798, GC_00004803, GC_00020322, GC_00023954	0.6	1.0	Bradyrhizobium
spermidine synthase [EC:2.5.1.16]	4.62	0.032	1.00	control	K00797	GC_00004761, GC_00004893, GC_00015455, GC_00020520	0.6	1.0	Bradyrhizobium
cell filamentation protein, protein adenylyltransferase [EC:2.7.7.10	4.62	0.032	1.00	control	K04095	GC_00006262, GC_00010315, GC_00010399, GC_00010634, GC_00012206, GC_00013808, GC_00015965, GC_00021162, GC_00021214, GC_00022608, GC_00026007	0.6	1.0	Bradyrhizobium
pimeloyl-[acyl-carrier protein] methyl ester esterase [EC:3.1.1.85]	4.62	0.032	1.00	control	K02170	GC_00003531	0.6	1.0	Bradyrhizobium
bifunctional enzyme CysN/CysC [EC:2.7.7.4 2.7.1.25]	4.26	0.039	1.00	heated	K00955	GC_00007761	0.6	0.1	Bradyrhizobium
diphthine-ammonia ligase [EC:6.3.1.14]	4.26	0.039	1.00	heated	K06927	GC_00007675	0.6	0.1	Bradyrhizobium
porin	4.26	0.039	1.00	heated	K07267	GC_00012483, GC_00013359, GC_00015225, GC_00017427, GC_00017871, GC_00021685	0.6	0.1	Bradyrhizobium
ribosome-dependent ATPase	4.26	0.039	1.00	heated	K13926	GC_00006975	0.6	0.1	Bradyrhizobium
restriction system protein	4.26	0.039	1.00	heated	K07448	GC_00013073, GC_00014310, GC_00020243, GC_00024998	0.6	0.1	Bradyrhizobium
cobalamin biosynthesis protein CobC	4.26	0.039	1.00	control	K02225	GC_00006016, GC_00009971, GC_00012944	0.4	0.9	Bradyrhizobium
alpha-terpineol hydroxylase	4.26	0.039	1.00	control	K24391	GC_00004166	0.4	0.9	Bradyrhizobium
antitoxin Phd	7.78	0.005	0.37	control	K19165	GC_00005733, GC_00006702, GC_00006805	0.0	0.8	Rhizobium
para-nitrobenzyl esterase [EC:3.1.1.-]	7.78	0.005	0.37	control	K03929	GC_00004995, GC_00005404, GC_00005681	0.0	0.8	Rhizobium
FAD-dependent urate hydroxylase [EC:1.14.13.113]	7.78	0.005	0.37	control	K22879	GC_00004951	0.0	0.8	Rhizobium
LysR family transcriptional regulator, benzoate and cis,cis-muconate-responsive activator of ben and cat genes	7.78	0.005	0.37	control	K21757	GC_00005208, GC_00006886	0.0	0.8	Rhizobium
cell filamentation protein, protein adenylyltransferase [EC:2.7.7.10	7.78	0.005	0.37	control	K04095	GC_00005084, GC_00005477, GC_00006353, GC_00006468, GC_00006890, GC_00007645	0.0	0.8	Rhizobium
alanine dehydrogenase [EC:1.4.1.1]	7.78	0.005	0.37	control	K19244	GC_00005227, GC_00006715	0.0	0.8	Rhizobium
gamma-glutamylcyclotransferase [EC:4.3.2.9]	7.78	0.005	0.37	heated	K00682	GC_00004500	1.0	0.2	Rhizobium
RpiR family transcriptional regulator, repressor of rpiB and als oper	7.78	0.005	0.37	heated	K23238	GC_00004616	1.0	0.2	Rhizobium
TetR/AcrR family transcriptional regulator, repressor of the mexAB-oprM multidrug resistance operon	7.78	0.005	0.37	heated	K18135	GC_00004719	1.0	0.2	Rhizobium
alcohol dehydrogenase [EC:1.1.1.-]	7.78	0.005	0.37	heated	K18369	GC_00004556	1.0	0.2	Rhizobium
cobalt-zinc-cadmium efflux system protein	7.78	0.005	0.37	heated	K16264	GC_00004825	1.0	0.2	Rhizobium
primary-amine oxidase [EC:1.4.3.21]	7.78	0.005	0.37	heated	K00276	GC_00004899	1.0	0.2	Rhizobium
tellurite methyltransferase [EC:2.1.1.265]	7.78	0.005	0.37	heated	K16868	GC_00004640	1.0	0.2	Rhizobium
DNA sulfur modification protein DndE	7.78	0.005	0.37	heated	K19172	GC_00004722	1.0	0.2	Rhizobium
MFS transporter, DHA1 family, multidrug resistance protein B	7.78	0.005	0.37	heated	K08152	GC_00002364	1.0	0.2	Rhizobium

Table S4. KOfam Functional Enrichment

Description	Enrichment Score	P-value	Q-value	Associated Treatment	KO Identifier	Gene Cluster IDs	p_Heated	p_Control	Clade
3-hydroxy-9,10-secoandrosta-1,3,5(10)-triene-9,17-dione monooxygenase [EC:1.14.14.12]	7.78	0.005	0.37	heated	K16047	GC_00001250	1.0	0.2	Rhizobium
glucose dehydrogenase [EC:1.1.5.9]	7.78	0.005	0.37	heated	K19813	GC_00004917	1.0	0.2	Rhizobium
DNA sulfur modification protein DndD	7.78	0.005	0.37	heated	K19171	GC_00003866	1.0	0.2	Rhizobium
dimethylamine monooxygenase subunit B [EC:1.14.13.238]	7.78	0.005	0.37	heated	K22343	GC_00004797	1.0	0.2	Rhizobium
trimethylamine monooxygenase [EC:1.14.13.148]	7.78	0.005	0.37	heated	K18277	GC_00004889	1.0	0.2	Rhizobium
DNA sulfur modification protein DndB	7.78	0.005	0.37	heated	K19169	GC_00004337, GC_00004549	1.0	0.2	Rhizobium
limonene 1,2-monooxygenase [EC:1.14.13.107]	7.78	0.005	0.37	heated	K14733	GC_00004418	1.0	0.2	Rhizobium
dimethylamine monooxygenase subunit C [EC:1.14.13.238]	7.78	0.005	0.37	heated	K22344	GC_00004781	1.0	0.2	Rhizobium
N,N-dimethylformamidase large subunit [EC:3.5.1.56]	7.78	0.005	0.37	heated	K03418	GC_00004675	1.0	0.2	Rhizobium
RNA-directed DNA polymerase [EC:2.7.7.49]	7.78	0.005	0.37	heated	K00986	GC_00004998	1.0	0.2	Rhizobium
fructose 5-dehydrogenase cytochrome subunit	7.78	0.005	0.37	heated	K23275	GC_00004878	1.0	0.2	Rhizobium
DNA sulfur modification protein DndC	7.78	0.005	0.37	heated	K19170	GC_00003922	1.0	0.2	Rhizobium
4,5-dihydroxyphthalate decarboxylase [EC:4.1.1.55]	7.78	0.005	0.37	heated	K04102	GC_00003733	1.0	0.2	Rhizobium
dimethylamine monooxygenase subunit A [EC:1.14.13.238]	7.78	0.005	0.37	heated	K22342	GC_00004841	1.0	0.2	Rhizobium
protein-tyrosine phosphatase [EC:3.1.3.48]	7.78	0.005	0.37	heated	K01104	GC_00004710	1.0	0.2	Rhizobium
fructose 5-dehydrogenase small subunit [EC:1.1.5.14]	7.78	0.005	0.37	heated	K23274	GC_00004906	1.0	0.2	Rhizobium
DNA-binding protein HU-beta	6.64	0.010	0.68	control	K03530	GC_00004426, GC_00005335	0.2	0.9	Rhizobium
Rrf2 family transcriptional regulator, nitric oxide-sensitive transcriptional repressor	5.83	0.016	0.77	heated	K13771	GC_00004586	1.0	0.3	Rhizobium
aspartate 1-decarboxylase [EC:4.1.1.11]	5.83	0.016	0.77	heated	K01579	GC_00004562	1.0	0.3	Rhizobium
5-methylcytosine-specific restriction enzyme A [EC:3.1.21.-]	5.83	0.016	0.77	heated	K07451	GC_00003918, GC_00007265, GC_00007760	1.0	0.3	Rhizobium
peptidyl carrier protein	5.83	0.016	0.77	heated	K21183	GC_00004498	1.0	0.3	Rhizobium
DNA mismatch endonuclease, patch repair protein [EC:3.1.-.-]	5.83	0.016	0.77	heated	K07458	GC_00004580	1.0	0.3	Rhizobium
MbtH protein	5.83	0.016	0.77	heated	K05375	GC_00004590	1.0	0.3	Rhizobium
D-glycero-D-manno-heptose 1,7-bisphosphate phosphatase [EC:3.1.3.82 3.1.3.83]	5.83	0.016	0.77	control	K03273	GC_00005376, GC_00006750	0.0	0.7	Rhizobium
Bacteriophage probable baseplate hub protein	5.83	0.016	0.77	control	K06905	GC_00005334, GC_00006740	0.0	0.7	Rhizobium
mRNA interferase HigB [EC:3.1.-.-]	4.38	0.036	0.77	heated	K19166	GC_00005049	0.8	0.2	Rhizobium
cyclohexyl-isocyanide hydratase [EC:4.2.1.103]	4.32	0.038	0.77	heated	K18199	GC_00004551	1.0	0.4	Rhizobium
FAD:protein FMN transferase [EC:2.7.1.180]	4.32	0.038	0.77	heated	K03734	GC_00004153	1.0	0.4	Rhizobium
alginate O-acetyltransferase complex protein AlgI	4.32	0.038	0.77	heated	K19294	GC_00004548	1.0	0.4	Rhizobium
filamentous hemagglutinin	4.32	0.038	0.77	heated	K15125	GC_00000253	1.0	0.4	Rhizobium
limonene-1,2-epoxide hydrolase [EC:3.3.2.8]	4.32	0.038	0.77	heated	K10533	GC_00004522	1.0	0.4	Rhizobium
LuxR family transcriptional regulator, activator of conjugal transfer of Ti plasmids	4.32	0.038	0.77	heated	K19732	GC_00004631, GC_00006943	1.0	0.4	Rhizobium
mannuronan 5-epimerase [EC:5.1.3.37]	4.32	0.038	0.77	heated	K01795	GC_00000274	1.0	0.4	Rhizobium
5-(hydroxymethyl)furfural/furfural oxidase [EC:1.1.3.47 1.1.3.-]	4.32	0.038	0.77	heated	K16873	GC_00004392	1.0	0.4	Rhizobium
GDP-mannose 6-dehydrogenase [EC:1.1.1.132]	4.32	0.038	0.77	heated	K00066	GC_00004521	1.0	0.4	Rhizobium
death on curing protein	4.32	0.038	0.77	heated	K07341	GC_00004476	1.0	0.4	Rhizobium

Table S4. KOfam Functional Enrichment

Description	Enrichment Score	P-value	Q-value	Associated Treatment	KO Identifier	Gene Cluster IDs	p_Heated	p_Control	Clade
TraR antiactivator	4.32	0.038	0.77	heated	K20272	GC_00004748, GC_00006213	1.0	0.4	Rhizobium
competence protein ComFC	4.32	0.038	0.77	heated	K02242	GC_00004545	1.0	0.4	Rhizobium
acyl homoserine lactone synthase [EC:2.3.1.184]	4.32	0.038	0.77	heated	K22955	GC_00004249	1.0	0.4	Rhizobium
inhibitor of KinA	4.32	0.038	0.77	heated	K06351	GC_00004683, GC_00006346	1.0	0.4	Rhizobium
type IV secretion system protein TrbH	4.32	0.038	0.77	heated	K20267	GC_00004449	1.0	0.4	Rhizobium
mannuronan synthase [EC:2.4.1.33]	4.32	0.038	0.77	heated	K19290	GC_00003953, GC_00004326	1.0	0.4	Rhizobium
alginate O-acetyltransferase complex protein AlgJ	4.32	0.038	0.77	heated	K19295	GC_00004465	1.0	0.4	Rhizobium
type IV secretion system protein TrbG	4.32	0.038	0.77	heated	K20532	GC_00004401	1.0	0.4	Rhizobium
periplasmic protein CpxP/Spy	4.32	0.038	0.77	heated	K06006	GC_00004380	1.0	0.4	Rhizobium
DNA (cytosine-5)-methyltransferase 1 [EC:2.1.1.37]	4.32	0.038	0.77	heated	K00558	GC_00004599, GC_00004666, GC_00004941, GC_00004961, GC_00007065, GC_00007853	1.0	0.4	Rhizobium
5'-nucleotidase [EC:3.1.3.5]	4.32	0.038	0.77	heated	K02566	GC_00000965	1.0	0.4	Rhizobium
type IV secretion system protein TrbC	4.32	0.038	0.77	heated	K20528	GC_00004421, GC_00007376	1.0	0.4	Rhizobium
hemolysin activation/secretion protein	4.32	0.038	0.77	heated	K07326	GC_00003970	1.0	0.4	Rhizobium
large repetitive protein	4.32	0.038	0.77	heated	K20276	GC_00004422, GC_00006445	1.0	0.4	Rhizobium
2,5-diketo-D-gluconate reductase A [EC:1.1.1.346]	4.32	0.038	0.77	heated	K06221	GC_00004475	1.0	0.4	Rhizobium
TetR/AcrR family transcriptional regulator, cholesterol catabolism regulator	4.32	0.038	0.77	heated	K22107	GC_00004524	1.0	0.4	Rhizobium
alginate O-acetyltransferase complex protein AlgF	4.32	0.038	0.77	heated	K19296	GC_00004373	1.0	0.4	Rhizobium
alginate biosynthesis protein AlgX	4.32	0.038	0.77	heated	K19293	GC_00004437	1.0	0.4	Rhizobium
DNA-damage-inducible protein J	4.32	0.038	0.77	heated	K07473	GC_00000546	1.0	0.4	Rhizobium
type IV secretion system protein TrbK	4.32	0.038	0.77	heated	K20555	GC_00004756, GC_00006167	1.0	0.4	Rhizobium
aspartate-semialdehyde dehydrogenase [EC:1.2.1.-]	4.32	0.038	0.77	control	K15786	GC_00005174	0.0	0.6	Rhizobium
tRNA (cmo5U34)-methyltransferase [EC:2.1.1.-]	4.32	0.038	0.77	control	K15256	GC_00005096, GC_00005337	0.0	0.6	Rhizobium
type IV secretion system protein VirB4 [EC:7.4.2.8]	4.32	0.038	0.77	control	K03199	GC_00005112	0.0	0.6	Rhizobium
arylformamidase [EC:3.5.1.9]	4.32	0.038	0.77	control	K07130	GC_00005162, GC_00005447, GC_00006745	0.0	0.6	Rhizobium
type IV secretion system protein VirB9	4.32	0.038	0.77	control	K03204	GC_00005255	0.0	0.6	Rhizobium
type IV secretion system protein VirB7	4.32	0.038	0.77	control	K03202	GC_00005142	0.0	0.6	Rhizobium
2-dehydro-3-deoxy-L-rhamnonate dehydrogenase (NAD+) [EC:1.1.1.401]	4.32	0.038	0.77	control	K21883	GC_00005163, GC_00006435	0.0	0.6	Rhizobium
ectoine hydrolase [EC:3.5.4.44]	4.32	0.038	0.77	control	K15783	GC_00005157	0.0	0.6	Rhizobium
mannopine transport system substrate-binding protein	4.32	0.038	0.77	control	K11077	GC_00005262	0.0	0.6	Rhizobium
type IV secretion system protein VirB3	4.32	0.038	0.77	control	K03198	GC_00005115	0.0	0.6	Rhizobium
chromate reductase, NAD(P)H dehydrogenase (quinone)	4.32	0.038	0.77	control	K19784	GC_00005218	0.0	0.6	Rhizobium
aldehyde dehydrogenase (NAD+) [EC:1.2.1.3]	4.32	0.038	0.77	control	K00128	GC_00004655	0.0	0.6	Rhizobium
mannopine transport system permease protein	4.32	0.038	0.77	control	K11078	GC_00005173, GC_00005176	0.0	0.6	Rhizobium
type IV secretion system protein VirB10	4.32	0.038	0.77	control	K03195	GC_00005230	0.0	0.6	Rhizobium
GntR family transcriptional regulator, colanic acid and biofilm gene transcriptional regulator	4.32	0.038	0.77	control	K13654	GC_00005150	0.0	0.6	Rhizobium
simple sugar transport system ATP-binding protein [EC:7.5.2.-]	4.32	0.038	0.77	control	K02056	GC_00005144	0.0	0.6	Rhizobium

Table S4. KOfam Functional Enrichment

Description	Enrichment Score	P-value	Q-value	Associated Treatment	KO Identifier	Gene Cluster IDs	p_Heated	p_Control	Clade
putative Mg2+ transporter-C (MgtC) family protein	4.32	0.038	0.77	control	K07507	GC_00005152	0.0	0.6	Rhizobium
type IV secretion system protein VirB6	4.32	0.038	0.77	control	K03201	GC_00005147	0.0	0.6	Rhizobium
type IV secretion system protein VirB1	4.32	0.038	0.77	control	K03194	GC_00005128	0.0	0.6	Rhizobium
IclR family transcriptional regulator, blcABC operon repressor	4.32	0.038	0.77	control	K20539	GC_00005178	0.0	0.6	Rhizobium
mannopine transport system ATP-binding protein	4.32	0.038	0.77	control	K11080	GC_00005161	0.0	0.6	Rhizobium
transposase, IS30 family	4.32	0.038	0.77	control	K07482	GC_00005650, GC_00006268	0.0	0.6	Rhizobium
type IV secretion system protein VirB11 [EC:7.4.2.8]	4.32	0.038	0.77	control	K03196	GC_00005046	0.0	0.6	Rhizobium
8-hydroxy-5-deazaflavin:NADPH oxidoreductase [EC:1.5.1.40]	4.32	0.038	0.77	control	K06988	GC_00005793, GC_00006394	0.0	0.6	Rhizobium
N2-acetyl-L-2,4-diaminobutanoate deacetylase [EC:3.5.1.125]	4.32	0.038	0.77	control	K15784	GC_00005195	0.0	0.6	Rhizobium
type IV secretion system protein VirB2	4.32	0.038	0.77	control	K03197	GC_00005098	0.0	0.6	Rhizobium
type IV secretion system protein VirB8	4.32	0.038	0.77	control	K03203	GC_00005103	0.0	0.6	Rhizobium
antitoxin FitA	4.32	0.038	0.77	control	K21495	GC_00005093	0.0	0.6	Rhizobium
DeoR family transcriptional regulator, deoxyribose operon represso	4.32	0.038	0.77	control	K11534	GC_00004672	0.0	0.6	Rhizobium
type IV secretion system protein VirB5	4.32	0.038	0.77	control	K03200	GC_00005229	0.0	0.6	Rhizobium
glutamate dehydrogenase (NADP+) [EC:1.4.1.4]	4.32	0.038	0.77	control	K00262	GC_00005253	0.0	0.6	Rhizobium
regulator of nucleoside diphosphate kinase	4.32	0.038	0.77	control	K06140	GC_00005102, GC_00005119	0.0	0.6	Rhizobium
3-demethoxyubiquinol 3-hydroxylase [EC:1.14.99.60]	4.32	0.038	0.77	control	K06134	GC_00005231	0.0	0.6	Rhizobium
D-alanyl-D-alanine carboxypeptidase [EC:3.4.16.4]	4.32	0.038	0.77	control	K01286	GC_00005584, GC_00006158	0.0	0.6	Rhizobium
L-2,4-diaminobutyrate transaminase [EC:2.6.1.76]	4.32	0.038	0.77	control	K15785	GC_00005082	0.0	0.6	Rhizobium
SlyX protein	4.20	0.040	0.80	control	K03745	GC_00003990	0.6	1.0	Rhizobium
chorismate mutase [EC:5.4.99.5]	4.20	0.040	0.80	control	K04092	GC_00004002, GC_00005864	0.6	1.0	Rhizobium
large subunit ribosomal protein L27	4.20	0.040	0.80	control	K02899	GC_00003987	0.6	1.0	Rhizobium
flagellar biosynthesis protein	4.20	0.040	0.80	heated	K04061	GC_00006076	0.4	0.0	Rhizobium
antitoxin HigA-1	8.12	0.004	1.00	heated	K21498	GC_00004018, GC_00004720, GC_00010539, GC_00010540, GC_00017723, GC_00022049, GC_00024334	1.0	0.4	Paraburkholderia
3-oxoisoapionate-4-phosphate transcarboxylase/hydrolase [EC:3.7.1.28]	8.12	0.004	1.00	control	K01559	GC_00011190, GC_00013200	0.0	0.6	Paraburkholderia
antitoxin ParD1/3/4	8.12	0.004	1.00	control	K07746	GC_00011027, GC_00022627	0.0	0.6	Paraburkholderia
3-oxoisoapionate kinase [EC:2.7.1.231]	8.12	0.004	1.00	control	K23247	GC_00011463, GC_00013087	0.0	0.6	Paraburkholderia
acrylyl-CoA reductase (NADPH) [EC:1.3.1.-]	8.12	0.004	1.00	control	K19745	GC_00008334	0.0	0.6	Paraburkholderia
aromatic-L-amino-acid/L-tryptophan decarboxylase [EC:4.1.1.28 4.1.1.105]	8.12	0.004	1.00	control	K01593	GC_00011166, GC_00012791	0.0	0.6	Paraburkholderia
LuxR family transcriptional regulator, transcriptional regulator of spore coat protein	8.04	0.005	1.00	control	K01994	GC_00008552, GC_00015838	0.1	0.8	Paraburkholderia
carnitine-CoA ligase [EC:6.2.1.48]	5.66	0.017	1.00	control	K02182	GC_00008118, GC_00010237, GC_00012914, GC_00019298, GC_00021041, GC_00022782, GC_00024366	0.4	1.0	Paraburkholderia
alpha-galactosidase [EC:3.2.1.22]	5.60	0.018	1.00	control	K07407	GC_00007528, GC_00022964	0.2	0.8	Paraburkholderia
toxin CcdB	5.60	0.018	1.00	control	K19163	GC_00009832, GC_00012944, GC_00021954	0.2	0.8	Paraburkholderia
ATP-dependent Clp protease ATP-binding subunit ClpC	5.60	0.018	1.00	control	K03696	GC_00007410, GC_00008183	0.2	0.8	Paraburkholderia

Table S4. KOfam Functional Enrichment

Description	Enrichment Score	P-value	Q-value	Associated Treatment	KO Identifier	Gene Cluster IDs	p_Heated	p_Control	Clade
cation:H+ antiporter	5.60	0.018	1.00	control	K07301	GC_00006657	0.2	0.8	Paraburkholderia
antitoxin CcdA	5.60	0.018	1.00	control	K19164	GC_00009506, GC_00009507, GC_00018685, GC_00020449	0.2	0.8	Paraburkholderia
lysozyme [EC:3.2.1.17]	5.60	0.018	1.00	control	K01185	GC_00010956, GC_00012539, GC_00014544, GC_00014860, GC_00017216, GC_00019240	0.2	0.8	Paraburkholderia
deoxyribodipyrimidine photo-lyase [EC:4.1.99.3]	5.03	0.025	1.00	heated	K01669	GC_00003501	1.0	0.6	Paraburkholderia
putative colanic acid biosynthesis glycosyltransferase WcaI	5.03	0.025	1.00	heated	K03208	GC_00000853, GC_00024058	1.0	0.6	Paraburkholderia
alkyldihydroxyacetonephosphate synthase [EC:2.5.1.26]	5.03	0.025	1.00	control	K00803	GC_00013098	0.0	0.4	Paraburkholderia
3-oxocholest-4-en-26-oyl-CoA dehydrogenase beta subunit [EC:1.3.99.-]	5.03	0.025	1.00	control	K22819	GC_00010244	0.0	0.4	Paraburkholderia
membrane fusion protein, type I secretion system	5.03	0.025	1.00	control	K12537	GC_00013006	0.0	0.4	Paraburkholderia
MarR family transcriptional regulator, 2-MHQ and catechol-resistance regulon repressor	5.03	0.025	1.00	control	K15973	GC_00011401	0.0	0.4	Paraburkholderia
4-hydroxybutyrate dehydrogenase / sulfolactaldehyde 3-reductase [EC:1.1.1.61 1.1.1.373]	5.03	0.025	1.00	control	K08318	GC_00011856	0.0	0.4	Paraburkholderia
3,4-dihydroxy-9,10-secoandrosta-1,3,5(10)-triene-9,17-dione 4,5-dioxygenase [EC:1.13.11.25]	5.03	0.025	1.00	control	K16049	GC_00013586, GC_00018746	0.0	0.4	Paraburkholderia
D-apionate oxidoisomerase [EC:1.1.1.421]	5.03	0.025	1.00	control	K23245	GC_00012444	0.0	0.4	Paraburkholderia
ATP-binding cassette, subfamily C, type I secretion system permease/ATPase	5.03	0.025	1.00	control	K12536	GC_00013686	0.0	0.4	Paraburkholderia
N-acetylglucosaminyldiphosphoundecaprenol N-acetyl-beta-D-mannosaminyltransferase [EC:2.4.1.187]	5.03	0.025	1.00	control	K05946	GC_00012630	0.0	0.4	Paraburkholderia
3-hydroxybutyryl-CoA dehydratase [EC:4.2.1.55]	5.03	0.025	1.00	control	K17865	GC_00013298	0.0	0.4	Paraburkholderia
5-methylcytosine-specific restriction enzyme subunit McrC	5.03	0.025	1.00	control	K19147	GC_00016739, GC_00024226	0.0	0.4	Paraburkholderia
antitoxin FitA	5.03	0.025	1.00	control	K21495	GC_00015636, GC_00022972	0.0	0.4	Paraburkholderia
curli production protein	5.03	0.025	1.00	control	K04336	GC_00013201	0.0	0.4	Paraburkholderia
fumarate hydratase subunit beta [EC:4.2.1.2]	5.03	0.025	1.00	control	K01678	GC_00011596	0.0	0.4	Paraburkholderia
GntR family transcriptional regulator, sialic acid-inducible nan operon repressor	5.03	0.025	1.00	control	K22104	GC_00011398	0.0	0.4	Paraburkholderia
glycosyl transferase, family 25	5.03	0.025	1.00	control	K07270	GC_00012466	0.0	0.4	Paraburkholderia
5-oxoprolinase (ATP-hydrolysing) [EC:3.5.2.9]	5.03	0.025	1.00	control	K01469	GC_00012069	0.0	0.4	Paraburkholderia
fumarate hydratase subunit alpha [EC:4.2.1.2]	5.03	0.025	1.00	control	K01677	GC_00012745	0.0	0.4	Paraburkholderia
2,4-dienoyl-CoA reductase [(3E)-enoyl-CoA-producing], peroxisomal [EC:1.3.1.124]	5.03	0.025	1.00	control	K13237	GC_00011423, GC_00013011	0.0	0.4	Paraburkholderia
GntR family transcriptional regulator, glc operon transcriptional activator	5.03	0.025	1.00	control	K11474	GC_00012112	0.0	0.4	Paraburkholderia
sulfopyruvate decarboxylase subunit beta [EC:4.1.1.79]	5.03	0.025	1.00	control	K13039	GC_00010425	0.0	0.4	Paraburkholderia
dimethylsulfone monooxygenase [EC:1.14.14.35]	5.03	0.025	1.00	control	K17228	GC_00012582	0.0	0.4	Paraburkholderia
general L-amino acid transport system substrate-binding protein	5.03	0.025	1.00	control	K09969	GC_00009259, GC_00014341	0.0	0.4	Paraburkholderia
mannosyltransferase [EC:2.4.1.-]	5.03	0.025	1.00	control	K14340	GC_00013899, GC_00020125	0.0	0.4	Paraburkholderia
glycerol uptake operon antiterminator	5.03	0.025	1.00	control	K02443	GC_00012818	0.0	0.4	Paraburkholderia
sulfite dehydrogenase (cytochrome) subunit B [EC:1.8.2.1]	4.75	0.029	1.00	control	K00386	GC_00010228, GC_00021755	0.1	0.6	Paraburkholderia

Table S4. KOfam Functional Enrichment

Description	Enrichment Score	P-value	Q-value	Associated Treatment	KO Identifier	Gene Cluster IDs	p_Heated	p_Control	Clade
LytTR family transcriptional regulator, CO-responsive transcriptional regulator RcoM	4.75	0.029	1.00	control	K21696	GC_00008244	0.1	0.6	Paraburkholderia
2-oxopent-4-enoate/cis-2-oxohex-4-enoate hydratase [EC:4.2.1.80 4.2.1.132]	4.75	0.029	1.00	control	K18364	GC_00008491	0.1	0.6	Paraburkholderia
phosphatidylinositol-3-phosphatase [EC:3.1.3.64]	4.75	0.029	1.00	control	K21302	GC_00008634, GC_00023561	0.1	0.6	Paraburkholderia
zinc D-Ala-D-Ala dipeptidase [EC:3.4.13.22]	4.75	0.029	1.00	control	K08641	GC_00008012	0.1	0.6	Paraburkholderia
sulfite dehydrogenase (cytochrome) subunit A [EC:1.8.2.1]	4.75	0.029	1.00	control	K05301	GC_00008106	0.1	0.6	Paraburkholderia
salicylate 5-hydroxylase large subunit [EC:1.14.13.172]	4.75	0.029	1.00	control	K18242	GC_00007681	0.1	0.6	Paraburkholderia
TetR/AcrR family transcriptional regulator, mexJK operon transcriptional repressor	4.75	0.029	1.00	heated	K18301	GC_00004474, GC_00007246, GC_00012732, GC_00016990, GC_00018820	0.9	0.4	Paraburkholderia
acetoacetyl-CoA synthetase [EC:6.2.1.16]	4.75	0.029	1.00	heated	K01907	GC_00005396, GC_00010168, GC_00018859	0.9	0.4	Paraburkholderia
toxin HigB-1	4.75	0.029	1.00	heated	K07334	GC_00003962, GC_00008900, GC_00010510	0.9	0.4	Paraburkholderia
toxin CptA	4.75	0.029	1.00	heated	K19168	GC_00003930	0.9	0.4	Paraburkholderia
IclR family transcriptional regulator, mhp operon transcriptional activator	4.36	0.037	1.00	control	K05818	GC_00008314, GC_00008806, GC_00009979, GC_00011757, GC_00014364, GC_00016559, GC_00021690	0.5	1.0	Paraburkholderia
HTH-type transcriptional regulator, competence development regulator	4.36	0.037	1.00	heated	K22299	GC_00006163, GC_00010744	0.5	0.0	Paraburkholderia
mRNA interferase YafQ [EC:3.1.-.-]	4.36	0.037	1.00	heated	K19157	GC_00006181	0.5	0.0	Paraburkholderia
D-galactose 1-dehydrogenase [EC:1.1.1.48]	3.88	0.049	1.00	heated	K00035	GC_00004983	0.7	0.2	Paraburkholderia
citronellol/citronellal dehydrogenase	3.88	0.049	1.00	control	K13775	GC_00005054, GC_00007500, GC_00016332	0.3	0.8	Paraburkholderia
isobutyryl-CoA mutase [EC:5.4.99.13]	3.88	0.049	1.00	control	K11942	GC_00006141	0.3	0.8	Paraburkholderia
protein PsiE	3.88	0.049	1.00	control	K13256	GC_00007231, GC_00008902	0.3	0.8	Paraburkholderia
benzoate-CoA ligase [EC:6.2.1.25]	3.88	0.049	1.00	control	K04110	GC_00006098	0.3	0.8	Paraburkholderia
type IV secretion system protein TrbG	3.88	0.049	1.00	control	K20532	GC_00006581, GC_00014624	0.3	0.8	Paraburkholderia
choline-sulfatase [EC:3.1.6.6]	3.88	0.049	1.00	control	K01133	GC_00006116	0.3	0.8	Paraburkholderia
XRE family transcriptional regulator, aerobic/anaerobic benzoate catabolism transcriptional regulator	3.88	0.049	1.00	control	K15546	GC_00006001, GC_00017904	0.3	0.8	Paraburkholderia
molybdate transport system permease protein	3.88	0.049	1.00	control	K02018	GC_00005996	0.3	0.8	Paraburkholderia
benzoyl-CoA 2,3-epoxidase subunit A [EC:1.14.13.208]	3.88	0.049	1.00	control	K15511	GC_00006088	0.3	0.8	Paraburkholderia
ribulokinase [EC:2.7.1.16]	3.88	0.049	1.00	control	K24707	GC_00006147	0.3	0.8	Paraburkholderia
benzoyl-CoA-dihydrodiol lyase [EC:4.1.2.44]	3.88	0.049	1.00	control	K15513	GC_00006136	0.3	0.8	Paraburkholderia
type IV secretion system protein TrbF	3.88	0.049	1.00	control	K20531	GC_00006552, GC_00017401	0.3	0.8	Paraburkholderia
entericidin A	3.88	0.049	1.00	control	K16347	GC_00005951	0.3	0.8	Paraburkholderia
L-lactate dehydrogenase (cytochrome) [EC:1.1.2.3]	3.88	0.049	1.00	control	K00101	GC_00005524	0.3	0.8	Paraburkholderia
benzoyl-CoA 2,3-epoxidase subunit B [EC:1.14.13.208]	3.88	0.049	1.00	control	K15512	GC_00006056	0.3	0.8	Paraburkholderia
(S)-mandelate dehydrogenase [EC:1.1.99.31]	3.88	0.049	1.00	control	K15054	GC_00006560, GC_00009472, GC_00017903	0.3	0.8	Paraburkholderia

Table S5. KEGG Module Functional Enrichment

Description	Enrichment Score	P-value	Q-value	Associated Treatment	KEGG Identifier	Gene Cluster IDs	p_Heated	p_Control	Clade
Methanofuran biosynthesis	8.12	0.004	0.86	control	M00935	GC_00002980, GC_00017119	0.3	1.0	Kitasatospora
Nicotinate degradation, nicotinate => fumarate	7.02	0.008	0.86	control	M00622	GC_00005486, GC_00028536, GC_00028579	0.3	1.0	Kitasatospora
Calicheamicin biosynthesis, calicheamicinone => calicheamicin	7.02	0.008	0.86	heated	M00833	GC_00005614, GC_00005620, GC_00009704, GC_00016311, GC_00026255	0.7	0.0	Kitasatospora
Malonate semialdehyde pathway, propanoyl-CoA => acetyl-CoA;beta-Oxidation;beta-Oxidation, peroxisome, VLCFA;Jasmonic acid biosynthesis	6.39	0.011	0.87	control	M00013;M00087; M00861;M00113	GC_00007179, GC_00009968, GC_00013461, GC_00023709	0.2	0.8	Kitasatospora
Cysteine biosynthesis, serine => cysteine	6.09	0.014	0.87	heated	M00021	GC_00004630, GC_00025323, GC_00029039, GC_00029649	0.6	0.0	Kitasatospora
Fosfomycin biosynthesis, phosphoenolpyruvate => fosfomycin	5.38	0.020	0.91	control	M00903	GC_00010304, GC_00021033	0.1	0.6	Kitasatospora
Heparan sulfate degradation	5.20	0.023	0.91	control	M00078	GC_00005322	0.3	0.8	Kitasatospora
Homoprotocatechuate degradation, homoprotocatechuate => 2-oxohept-3-enedioate	5.20	0.023	0.91	heated	M00533	GC_00004867, GC_00015586, GC_00016351, GC_00018400, GC_00019132	0.7	0.2	Kitasatospora
Dihydrokalafungin biosynthesis, octaketide => dihydrokalafungin	4.77	0.029	1.00	heated	M00779	GC_00004048, GC_00006202, GC_00006714, GC_00011263, GC_00011682, GC_00012840, GC_00016375	1.0	0.8	Kitasatospora
Dissimilatory nitrate reduction, nitrate => ammonia	4.57	0.033	1.00	heated	M00530	GC_00004972, GC_00005380, GC_00020999	0.5	0.0	Kitasatospora
Betacyanin biosynthesis, L-tyrosine => amaranthin	4.23	0.040	1.00	control	M00961	GC_00010082, GC_00011523	0.3	0.8	Kitasatospora
Tryptophan metabolism, tryptophan => kynurenine => 2-aminomuconate	3.98	0.046	1.00	heated	M00038	GC_00004626, GC_00008818	0.8	0.4	Kitasatospora
Coenzyme M biosynthesis	3.94	0.047	1.00	heated	M00358	GC_00005869, GC_00008057	0.5	0.0	Kitasatospora
Assimilatory nitrate reduction, nitrate => ammonia;Nitrate assimilation	3.94	0.047	1.00	heated	M00531;M00615	GC_00005201, GC_00005371	0.5	0.0	Kitasatospora
Ubiquinone biosynthesis, prokaryotes, chorismate (+ polyprenyl-PP) => ubiquinol;C5 isoprenoid biosynthesis, mevalonate pathway, archaea	5.00	0.025	1.00	heated	M00117;M00849	GC_00006040, GC_00009484, GC_00010750	0.8	0.2	Bradyrhizobium
Pimeloyl-ACP biosynthesis, BioC-BioH pathway, malonyl-ACP => pimeloyl-ACP	4.62	0.032	1.00	control	M00572	GC_00003531	0.6	1.0	Bradyrhizobium
Methionine salvage pathway;Polyamine biosynthesis, arginine => agmatine => putrescine => spermidine	4.62	0.032	1.00	control	M00034;M00133	GC_00004761, GC_00004893, GC_00015455, GC_00020520	0.6	1.0	Bradyrhizobium
GABA (gamma-Aminobutyrate) shunt	4.26	0.039	1.00	heated	M00027	GC_00010877, GC_00012424, GC_00025959	0.6	0.1	Bradyrhizobium
Phthalate degradation, phthalate => protocatechuate	7.78	0.005	0.77	heated	M00623	GC_00003733	1.0	0.2	Rhizobium
Pantothenate biosynthesis, valine/L-aspartate => pantothenate	5.83	0.016	0.77	heated	M00119	GC_00004562	1.0	0.3	Rhizobium

Table S5. KEGG Module Functional Enrichment

Description	Enrichment Score	P-value	Q-value	Associated Treatment	KEGG Identifier	Gene Cluster IDs	p_Heated	p_Control	Clade
C-1027 beta-amino acid moiety biosynthesis, tyrosine => 3-chloro-4,5-dihydroxy-beta-phenylalanyl-PCP;Maduropeptin beta-hydroxy acid moiety biosynthesis, tyrosine => 3-(4-hydroxyphenyl)-3-oxopropanoyl-PCP;Kedarcidin 2-aza-3-chloro-beta-tyrosine moiety biosynthesis, azatyrosine => 2-aza-3-chloro-beta-tyrosyl-PCP	5.83	0.016	0.77	heated	M00827;M00828; M00832	GC_00004498	1.0	0.3	Rhizobium
Nocardicin A biosynthesis, L-pHPG + arginine + serine => nocardicin A	5.83	0.016	0.77	heated	M00736	GC_00004590	1.0	0.3	Rhizobium
ADP-L-glycero-D-manno-heptose biosynthesis	5.83	0.016	0.77	control	M00064	GC_00005369, GC_00005374, GC_00005376, GC_00006750, GC_00006849	0.0	0.7	Rhizobium
Ubiquinone biosynthesis, prokaryotes, chorismate (+ polyprenyl-PP) => ubiquinol;Ubiquinone biosynthesis, eukaryotes, 4-hydroxybenzoate + polyprenyl-PP => ubiquinol	4.32	0.038	0.90	control	M00117;M00128	GC_00005231	0.0	0.6	Rhizobium
Tryptophan metabolism, tryptophan => kynurenine => 2-aminomuconate;NAD biosynthesis, tryptophan => quinolinate => NAD	4.32	0.038	0.90	control	M00038;M00912	GC_00005162, GC_00005447, GC_00005773, GC_00006745	0.0	0.6	Rhizobium
GABA biosynthesis, eukaryotes, putrescine => GABA;Pantothenate biosynthesis, 2-oxoisovalerate/spermine => pantothenate	4.32	0.038	0.90	control	M00135;M00913	GC_00004655	0.0	0.6	Rhizobium
Ectoine degradation, ectoine => aspartate	4.32	0.038	0.90	control	M00919	GC_00005082, GC_00005157, GC_00005174, GC_00005195	0.0	0.6	Rhizobium
Helicobacter pylori pathogenicity signature, cagA pathogenicity island	4.32	0.038	0.90	control	M00564	GC_00005046	0.0	0.6	Rhizobium
Phenylalanine biosynthesis, chorismate => phenylpyruvate => phenylalanine;Tyrosine biosynthesis, chorismate => HPP => tyrosine;Tyrosine biosynthesis, chorismate => arogenate => tyrosine	4.20	0.040	0.90	control	M00024;M00025; M00040	GC_00004002, GC_00005864	0.6	1.0	Rhizobium
Catecholamine biosynthesis, tyrosine => dopamine => noradrenaline => adrenaline;Melatonin biosynthesis, animals, tryptophan => serotonin => melatonin;Melatonin biosynthesis, plants, tryptophan => serotonin => melatonin	8.12	0.004	0.73	control	M00042;M00037; M00936	GC_00011166, GC_00012791	0.0	0.6	Paraburkholderia
Vancomycin resistance, D-Ala-D-Lac type	8.04	0.005	0.73	control	M00651	GC_00008012, GC_00017324, GC_00022669	0.1	0.8	Paraburkholderia
Citrate cycle (TCA cycle, Krebs cycle);Citrate cycle, second carbon oxidation, 2-oxoglutarate => oxaloacetate;Reductive citrate cycle (Arnon-Buchanan cycle);Dicarboxylate-hydroxybutyrate cycle;Incomplete reductive citrate cycle, acetyl-CoA => oxoglutarate;Anoxygenic photosynthesis in green nonsulfur bacteria;Anoxygenic photosynthesis in green sulfur bacteria	5.03	0.025	1.00	control	M00009;M00011; M00173; M00374; M00620; M00613; M00614	GC_00011596, GC_00012745	0.0	0.4	Paraburkholderia
Ethylmalonyl pathway	5.03	0.025	1.00	control	M00373	GC_00013298	0.0	0.4	Paraburkholderia
Salicylate degradation, salicylate => gentisate	4.75	0.029	1.00	control	M00638	GC_00007681, GC_00008591	0.1	0.6	Paraburkholderia
Anoxygenic photosynthesis in green nonsulfur bacteria	3.88	0.049	1.00	control	M00613	GC_00006141	0.3	0.8	Paraburkholderia

Table S6. Metabolic Module Functional Enrichment

Description	Enrichment Score	P-value	Q-value	Associated Treatment	Module Identifier	Genomes	p_Heated	p_Control	Clade
Citrate cycle, first carbon oxidation, oxaloacetate => 2-oxoglutarate	9.91	0.002	0.10	heated	M00010	GP157, GP160, GP163, GP28, GP31, GP36, GP50, GP55, MAA19, MAA2, MAA52, MAA75, MAA81, MAP12-4, MAP12-9, MAP2-59, MAP5-34, MAP5-40, MAP8-42, GAS204B, MAP12-44, MAP12-15, MAA4, MAA18, GP30, GP82	1.0	0.6	Kitasatospora
Reductive citrate cycle (Arnon-Buchanan cycle)	9.91	0.002	0.10	heated	M00173	GP157, GP160, GP163, GP28, GP31, GP36, GP50, GP55, MAA19, MAA2, MAA52, MAA75, MAA81, MAP12-4, MAP12-9, MAP2-59, MAP5-34, MAP5-40, MAP8-42, GAS204B, MAP12-44, MAP12-15, MAA4, MAA18, GP30, GP82	1.0	0.6	Kitasatospora
Trans-cinnamate degradation, trans-cinnamate => acetyl-CoA	5.38	0.020	0.49	control	M00545	GAS1054, GP163, GP50, MAA52, MAA75, GAS204B	0.1	0.6	Kitasatospora
GABA biosynthesis, eukaryotes, putrescine => GABA	4.77	0.029	0.49	heated	M00135	GAS1054, GP157, GP160, GP163, GP28, GP31, GP36, GP50, GP55, MAA19, MAA2, MAA52, MAA75, MAA81, MAP12-4, MAP12-9, MAP25-9, MAP5-34, MAP5-40, MAP8-42, GAS204B, MAP124-4, MAP12-15, MAA4, MAA18, GP30, GP82	1.0	0.8	Kitasatospora
Glyoxylate cycle	4.77	0.029	0.49	heated	M00012	GP157, GP160, GP163, GP28, GP31, GP36, GP50, GP55, MAA19, MAA2, MAA36, MAA52, MAA75, MAA81, MAP12-4, MAP129, MAP2-59, MAP5-34, MAP5-40, MAP8-42, GAS204B, MAP12-44, MAP12-15, MAA4, MAA18, GP30, GP82	1.0	0.8	Kitasatospora
Pantothenate biosynthesis, 2-oxoisovalerate/spermine => pantothenate	4.77	0.029	0.49	heated	M00913	GAS1054, GP157, GP160, GP163, GP28, GP31, GP36, GP50, GP55, MAA19, MAA2, MAA52, MAA75, MAA81, MAP12-4, MAP12-9, MAP2-59, MAP5-34, MAP5-40, MAP8-42, GAS204B, MAP12-44, MAP12-15, MAA4, MAA18, GP30, GP82	1.0	0.8	Kitasatospora
beta-Oxidation	4.77	0.029	0.49	heated	M00087	GAS1054, GP157, GP160, GP163, GP28, GP31, GP36, GP50, GP55, MAA19, MAA2, MAA52, MAA75, MAA81, MAP12-4, MAP12-9, MAP2-59, MAP5-34, MAP5-40, MAP8-42, GAS204B, MAP12-44, MAP12-15, MAA4, MAA18, GP30, GP82	1.0	0.8	Kitasatospora
beta-Lactam resistance, Bla system	4.62	0.032	1.00	heated	M00627	GAS242, MAP5-43	0.4	0.0	Bradyrhizobium
Purine degradation, xanthine => urea	7.78	0.005	0.53	control	M00546	AN5, AN64, AN68, AN70, AN72, AN73, AN88	0.0	0.8	Rhizobium
Assimilatory nitrate reduction, nitrate => ammonia	6.64	0.010	0.53	control	M00531	AN5, AN64, AN68, AN69, AN6A, AN70, AN72, AN73, AN88	0.2	0.9	Rhizobium
Ectoine degradation, ectoine => aspartate	4.32	0.038	0.61	control	M00919	AN5, AN64, AN68, AN72, AN88	0.0	0.6	Rhizobium

Table S6. Metabolic Module Functional Enrichment

Description	Enrichment Score	P-value	Q-value	Associated Treatment	Module Identifier	Genomes	p_Heated	p_Control	Clade
Methionine degradation	4.32	0.038	0.61	heated	M00035	AN5, AN63, AN67, AN69, AN6A, AN72, AN83, AN95, 28DA2	1.0	0.4	Rhizobium
Pantothenate biosynthesis, 2-oxoisovalerate/spermine => pantothenate	4.32	0.038	0.61	control	M00913	AN64, AN68, AN70, AN73, AN88	0.0	0.6	Rhizobium
Cytochrome c oxidase, prokaryotes	4.20	0.040	0.61	control	M00155	AN5, AN63, AN64, AN67, AN68, AN69, AN6A, AN70, AN72, AN73, AN83, AN88	0.6	1.0	Rhizobium
Multidrug resistance, efflux pump MexJK-OprM	4.20	0.040	0.61	heated	M00642	AN63, AN83	0.4	0.0	Rhizobium

Table S7. CAZyme Functional Enrichment

CAZyme HMM	Enzyme Class	Family Activity Description	Substrate	Enrichment Score	P-value	Q-value	Associated Treatment	Gene Cluster IDs	p_Heated	p_Control	Clade
GH39.hmm	Glycoside hydrolases	3-O-β-L-arabinopyranosyl-α-L-arabinofuranosidase (EC 3.2.1.-); Endo-α-L-rhamnosidase (EC 3.2.1.-); β-glucosidase (EC 3.2.1.21); 3-O-α-D-galactosyl-α-L-arabinofuranosidase (EC 3.2.1.215); β-galactosidase (EC 3.2.1.23); Xylan β-1,4-xylosidase (EC 3.2.1.37); Exo-β-1,4-glucanase / cellodextrinase (EC 3.2.1.74); α-L-iduronidase (EC 3.2.1.76);	xylan, cellobiose, cellulose	6.39	0.011	0.73	control	GC_00007371, GC_00017393	0.2	0.8	Kitasatospora
GH19.hmm	Glycoside hydrolases	Chitinase (EC 3.2.1.14); Lysozyme (EC 3.2.1.17); [reducing end] exo-chitinase (EC 3.2.1.201);	chitin	5.28	0.022	0.73	control	GC_00003830	0.4	1.0	Kitasatospora
GH89.hmm	Glycoside hydrolases	α-N-acetylglucosaminidase (EC 3.2.1.50);		5.20	0.023	0.73	control	GC_00005322	0.3	0.8	Kitasatospora
GH13_21.hmm	Glycoside hydrolases	[retaining] α-amylase (EC 3.2.1.1); α-glucosidase (EC 3.2.1.20);	starch, glycogen	4.77	0.029	0.73	control	GC_00023147	0.0	0.2	Kitasatospora
PL31.hmm	Polysaccharide Lyases	Endo-β-1,4-glucuronan lyase (EC 4.2.2.14); Poly(β-mannuronate) lyase / M-specific alginate lyase (EC 4.2.2.3);	alginate	4.77	0.029	0.73	heated	GC_00000510, GC_00006367, GC_00019336	1.0	0.8	Kitasatospora
GH13_3.hmm	Glycoside hydrolases	α-1,4-glucan: phosphate α-maltosyltransferase (EC 2.4.99.16);		4.77	0.029	0.73	heated	GC_00000219	1.0	0.8	Kitasatospora
GH114.hmm	Glycoside hydrolases	[retaining] endo-α-1,4-galactosaminidase (EC 3.2.1.109);		4.57	0.033	0.73	control	GC_00004350, GC_00020399	0.5	1.0	Kitasatospora
GH119.hmm	Glycoside hydrolases	[retaining] α-amylase (EC 3.2.1.1);	starch	4.23	0.040	0.78	control	GC_00005030, GC_00013232	0.3	0.8	Kitasatospora
GH33.hmm	Glycoside hydrolases	Trans-sialidase (EC 2.4.1.-); 2-keto-3-deoxynononic acid hydrolase / KDNase (EC 3.2.1.-); Kdo hydrolase (EC 3.2.1.124); Exo-α-sialidase (EC 3.2.1.18); Anhydrosialidase (EC 4.2.2.15);		3.94	0.047	0.82	heated	GC_00005197, GC_00009621	0.5	0.0	Kitasatospora
GH74.hmm	Glycoside hydrolases	Oligoxyloglucan reducing-end-specific cellobiohydrolase (EC 3.2.1.150); Xyloglucan-specific endo-β-1,4-glucanase / endo-xyloglucanase (EC 3.2.1.151); Endo-β-1,4-glucanase (EC 3.2.1.4);	xyloglucan, cellulose	4.26	0.039	1.00	heated	GC_00010635, GC_00011244, GC_00017774, GC_00018033, GC_00018895, GC_00021200, GC_00025217	0.6	0.1	Bradyrhizobium
CE19.hmm	Carbhodrate Esterases	Pectin methylesterase (EC 3.1.1.11);		4.32	0.038	0.86	control	GC_00005263	0.6	0.0	Rhizobium

Table S7. CAZyme Functional Enrichment

CAZyme HMM	Enzyme Class	Family Activity Description	Substrate	Enrichment Score	P-value	Q-value	Associated Treatment	Gene Cluster IDs	p_Heated	p_Control	Clade
GT2_Glyco_tranf_2_3.hmm	Glycosyl-transferases	cellulose synthase (EC 2.4.1.12); chitin synthase (EC 2.4.1.16); dolichyl-phosphate beta-D-mannosyltransferase (EC 2.4.1.83); dolichyl-phosphate beta-glucosyltransferase (EC 2.4.1.117); N-acetylglucosaminyltransferase (EC 2.4.1.-); N-acetylgalactosaminyltransferase (EC 2.4.1.-); hyaluronan synthase (EC 2.4.1.212); chitin oligosaccharide synthase (EC 2.4.1.-); beta-1,3-glucan synthase (EC 2.4.1.34); beta-1,4-mannan synthase (EC 2.4.1.-); beta-mannosylphosphodecaprenol-mannooligosaccharide alpha-1,6-mannosyltransferase (EC 2.4.1.199); UDP-Galf: rhamnopyranosyl-N-acetylglucosaminyl-PP-decaprenol beta-1,4/1,5-galactofuranosyltransferase (EC 2.4.1.287); UDP-Galf: galactofuranosyl-galactofuranosyl-rhamnosyl-N-acetylglucosaminyl-PP-decaprenol beta-1,5/1,6-galactofuranosyltransferase (EC 2.4.1.288); dTDP-L-Rha: N-acetylglucosaminyl-PP-decaprenol alpha-1,3-L-rhamnosyltransferase (EC 2.4.1.289); alternating beta-1,3/4-N-acetylmannan synthase (2.4.1.-); UDP-GlcA: N-acetylglucosaminyl-proteoglycan beta-1,4-glucuronosyltransferase (EC 2.4.1.225); [inverting] UDP-Glc: glycocin S-beta-glucosyltransferase (EC 2.4.1.-); [inverting] UDP-Glc: protein O-beta-glucosyltransferase (EC 2.4.1.-)		4.32	0.038	0.86	heated	GC_00003953	0.4	1.0	Rhizobium
PL5.hmm	Polysaccharide Lyases	Endo-β-1,4-glucuronan lyase (EC 4.2.2.14); Poly(β-mannuronate) lyase / M-specific alginate lyase (EC 4.2.2.3);	alginate	4.32	0.038	0.86	heated	GC_00004263, GC_00004445	0.4	1.0	Rhizobium
GH24.hmm	Glycoside hydrolases	Lysozyme (EC 3.2.1.17);		5.60	0.018	0.74	control	GC_00010956, GC_00012539, GC_00014544, GC_00014860, GC_00017216, GC_00017413, GC_00019240	0.2	0.8	Paraburkholderia
GT25.hmm	Glycosyl-transferases	LPS β-1,4-N-acetylgalactosaminyltransferase (EC 2.4.1.-); UDP-Gal: β-1,4-galactosyltransferase (EC 2.4.1.-); UDP-Glc: hydroxylysine O-glucosyltransferase (EC 2.4.1.-); β-1,2-galactosyltransferase (EC 2.4.1.-); Occidiofungin β-xylosyltransferase (EC 2.4.2.-);		5.03	0.025	0.74	control	GC_00012466	0.0	0.4	Paraburkholderia
AA5.hmm	Auxiliary Activities	Raffinose oxidase (EC 1.1.3.-); Alcohol oxidase (EC 1.1.3.13); 5-(hydroxymethyl)furfural oxidase (EC 1.1.3.47); Aryl alcohol oxidase (EC 1.1.3.7); Galactose oxidase (EC 1.1.3.9); Glyoxal oxidase (EC 1.2.3.15);	raffinose, galactose	5.03	0.025	0.74	control	GC_00012899, GC_00020189, GC_00020567, GC_00024242	0.0	0.4	Paraburkholderia
GT26.hmm	Glycosyl-transferases	UDP-Gal: β-1,4-galactosyltransferase (EC 2.4.1.-); UDP-Glc: β-1,4-glucosyltransferase (EC 2.4.1.-); UDP-ManNAc: β-N-acetyl-mannosaminyltransferase (EC 2.4.1.-); UDP-ManNAcA: β-N-acetyl mannosaminuronyltransferase (EC 2.4.1.-);		5.03	0.025	0.74	control	GC_00012630	0.0	0.4	Paraburkholderia
GH92.hmm	Glycoside hydrolases	α-1,4-mannosidase (EC 3.2.1.-); Mannosyl-1-phosphodiester α-1,P-mannosidase (EC 3.2.1.-); Mannosyl-oligosaccharide α-1,3-mannosidase (EC 3.2.1.-); Mannosyl-oligosaccharide α-1,2-mannosidase (EC 3.2.1.113); α-mannosidase (EC 3.2.1.24);		3.88	0.049	0.74	heated	GC_00004487, GC_00024113	0.7	0.2	Paraburkholderia
